# Analysis of mobility data to build contact networks for COVID-19

**DOI:** 10.1371/journal.pone.0249726

**Published:** 2021-04-15

**Authors:** Katherine Klise, Walt Beyeler, Patrick Finley, Monear Makvandi

**Affiliations:** Sandia National Laboratories, Albuquerque, New Mexico, United States of America; University of Kentucky, UNITED STATES

## Abstract

As social distancing policies and recommendations went into effect in response to COVID-19, people made rapid changes to the places they visit. These changes are clearly seen in mobility data, which records foot traffic using location trackers in cell phones. While mobility data is often used to extract the number of customers that visit a particular business or business type, it is the frequency and duration of concurrent occupancy at those sites that governs transmission. Understanding the way people interact at different locations can help target policies and inform contact tracing and prevention strategies. This paper outlines methods to extract interactions from mobility data and build networks that can be used in epidemiological models. Several measures of interaction are extracted: interactions between people, the cumulative interactions for a single person, and cumulative interactions that occur at particular businesses. Network metrics are computed to identify structural trends which show clear changes based on the timing of stay-at-home orders. Measures of interaction and structural trends in the resulting networks can be used to better understand potential spreading events, the percent of interactions that can be classified as close contacts, and the impact of policy choices to control transmission.

## 1 Introduction

Transmission of COVID-19 is largely attributed to direct or close contact with infected individuals [1]. Indoor spaces with limited air exchange, recycled air, and a concentration of people have been shown to contribute to significant outbreaks [2, 3]. Social-distance policies have lowered the number of new cases, indicating that a general reduction in the number of contacts can decrease the number of individuals contracting the disease [4]. However, this wholesale reduction in contacts comes with high social and economic burdens [5]. Understanding the way people interact at different locations can help target policies, inform contact tracing, and develop prevention strategies as businesses reopen and social interactions increase.

Person-to-person interactions can be represented as a network that characterizes opportunities for disease transmission between members of a population. Networks that describe these interactions can be generated using a combination of statistical methods, census data, surveys, and mobility data (e.g., [6–13]). The prevalence of location tracking apps on cell phones produces a vast quantity of data that details the places that people visit and how long they stay at those locations. Recently, companies have stood up platforms such as the SafeGraph Data Consortium [14] and Google Community Mobility Reports [15] that provide device and aggregate mobility data to public officials and the research community to help respond to COVID-19. While device-level data tracks the mobility of individual cell phones, aggregation groups and anonymizes that data such that individual people cannot be identified.

As social distancing policies went into effect across the nation, people made rapid changes to the places they visit and the way they interact. Numerous news and journal articles have documented trends in mobility as social distance policies were put in place and then relaxed across different states (e.g., [16–20]). This type of analysis commonly uses metrics such as the number of visits, visit-hours, or the distance traveled to businesses as an indicator of traffic. While these metrics show clear trends that indicate policy changes decreased traffic, they do not capture the interactions that occur at these locations. For example, two businesses can have the same number of customers but different customer arrival times and durations. Simultaneous visits and clustering of individuals at a specific site are critical to understanding potential spreading events within a population.

Recent research by Chang et al. [6] use aggregate mobility data to build contact networks based on hourly visitor data to places of interest and time spent at home. The mobility data was combined with epidemiological models to calibrate transmission rates based on case counts for several cities in the United States. This approach illustrates that a small subset of places account for a large majority of infections and that disadvantaged groups are at higher risk of infection. Schlosser et al. [11] also coupled mobility data with epidemiological models and find that lockdown measures lead to more local, clustered networks which flatten the epidemic curve. Their research uses device-level mobility data and builds a network based on movement between counties in Germany. While certain populations might be underrepresented in mobility data, such as elderly, children, and prison populations [21], and mobility data typically focuses on places where commerce takes place [22], network analysis using this type of data can help identify trends in social behavior and guide policy decisions. Where mobility data is not available or unreliable, survey methods have also been used to build contact networks for the purpose of understanding disease transmission in smaller, underrepresented populations [13].

This paper presents methods to build contact networks from aggregate mobility data and quantifies changes in network structure and interactions that occur at specific places of interest over time. While several groups have proposed methods to generate contact networks from mobility data, the methods do not provide necessary information on spatial and temporal overlap of individuals at locations (e.g. [6, 11]). Our network generation method computes an interaction strength between individuals that is defined by the amount of time people simultaneously occupy the same business or home. This interaction strength is converted to a transmission rate for use in network informed epidemiological models, as described in Beyeler et al. [23]. In this paper, the networks are used in a series of epidemiological simulations to demonstrate how the methodology can be used to study closure policy. Results show that network structures change drastically in response to social distancing policies, with people having fewer, shorter interactions within more clustered groups. Results from the epidemiological simulations show that eliminating contacts prioritized by interaction strength can control disease spread.

## 2 Methods

**Fig 1:**
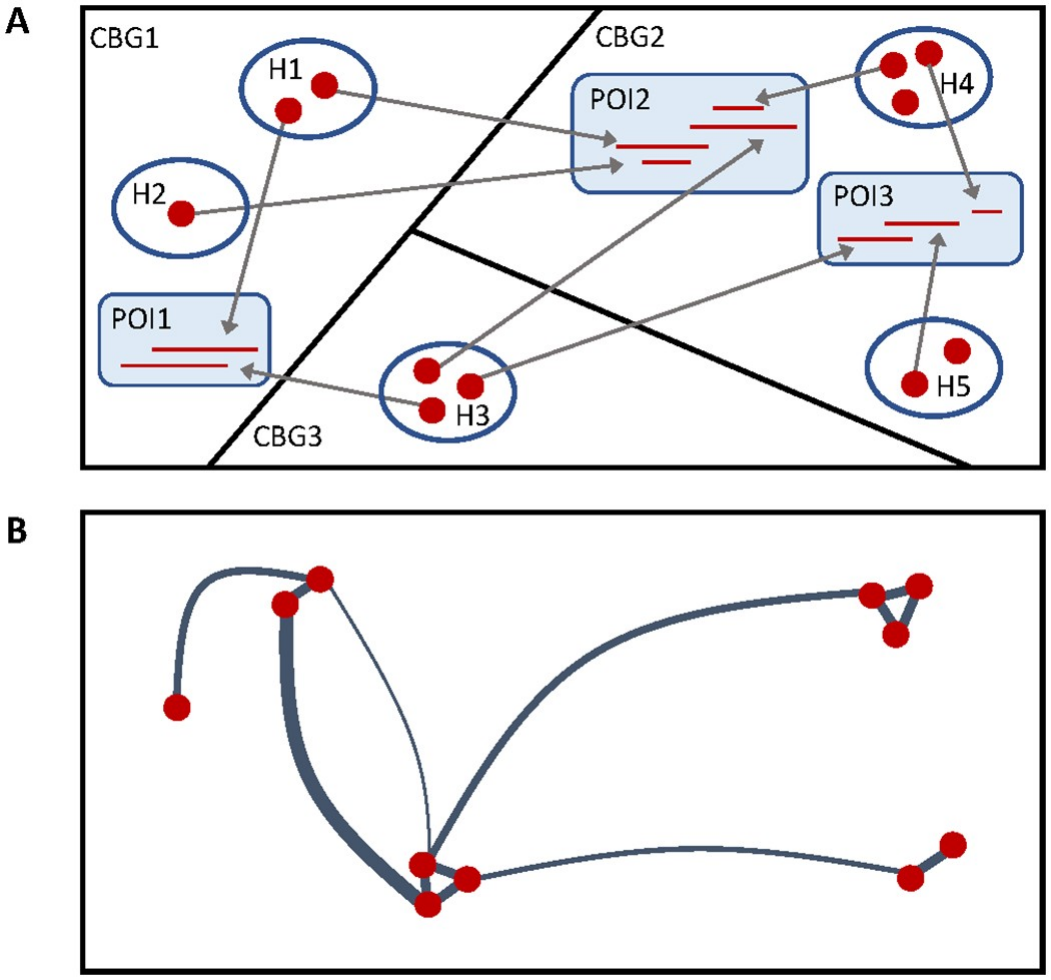
Diagram of contact network construction from mobility and census data. A. Mobility and census data represents people (red dots) from specific households (H) and census block groups (CBGs) visiting places of interest (POIs), B. Contact networks are generated from this data on a weekly basis. The edge weight represents the interaction strength between people.

This research uses aggregate mobility data published by SafeGraph [14] to create contact networks that represent person-to-person interactions. These interactions occur at specific places of interest (e.g., restaurants, schools, parks) and at home. The strength of interaction is based on arrival time and duration at places of interest and the amount of time spent at home. The network is created using a sampled population, which assigns people to home census block groups and clusters associated with households size and population based on census data. Visitors from outside the region of interest are not explicitly included in the contact network. The schematic diagram in Fig 1 illustrates the translation between mobility data and the contact network used in this paper. Fig 1A includes 3 census block groups (CBGs), 3 places of interest (POIs), and 11 people (red dots) grouped into 5 households (H). The red lines within each POI represent the arrival and departure time for each person that visits that POI. Person-to-person interactions occur when visits overlap in time, as shown in Fig 1B. The thickness of connecting edges represents the interaction strength between people. People within the same household interact according to the amount of concurrent time they spend at home.

The following steps are used to construct contact networks from mobility data. These steps, along with a description of the SafeGraph data, are addressed in the following subsections.

1. Sampling locations and visitor arrival times: To represent population sizes smaller than the area’s true population, scaling methods based on NAICS categories are used to constrain locations and visits.
2. Dwell time distributions: Dwell time is defined as the amount of time a visitor spends at a specific POI. While SafeGraph provides a coarse dwell time distribution, empirical distributions are created to approximate the median dwell time for each location.
3. Household contacts: Individuals are assigned to specific households based on household size and population associated with each CBG. Household contacts occur within these small clusters based on the amount of time spent at home within that CBG.
4. Concurrent occupancy: Concurrent occupancy occurs when people are in the same place at the same time. An interaction strength between individuals is defined from the arrival and dwell time distributions at POIs and time spent at home within each CBG.
5. Contact intensity: Because transmission risk is not the same at all places, variability of contact intensity can be included in the network construction.

The methods described below are used to construct contact networks at the county scale. The same methods have been used to create networks at other scales, for example at the city or state level. Bernalillo County in New Mexico is used to demonstrate the methods.

### 2.1 SafeGraph data

SafeGraph mobility data includes information about foot traffic at over 5 million places across the US based on cell phone records [14]. SafeGraph data focuses on locations were consumers can spend money and/or time. There are two exceptions to this rule, to include select industrial POIs and corporate offices for major organizations. The POIs in the database include individual schools, hospitals, parks, grocery stores, and restaurants, etc.

SafeGraph Weekly Patterns dataset [24] includes information about when people arrive at POIs, how long they stay, and where they came from. The data is anonymized by applying noise, omitting data associated with a single mobile device, and grouping traffic according to the home CBG of the mobile devices. The CBG is the highest resolution for census demographic information and generally contains between 600 to 3,000 people. Each POI in the SafeGraph database includes information on the number of devices that enter the POI on an hourly basis, a distribution of dwell times, and the device’s home CBG. SafeGraph determines the device’s home CBG by analyzing 6 weeks of data during nighttime hours (between 6 pm and 7 am). While not all devices can be assigned to a home location, a majority of the mobility data includes a home location. The Weekly Patterns data is used to define arrival and dwell time distributions to determine if people come into contact at POIs.

SafeGraph Social Distancing dataset [25] includes a timeseries that describes the number of devices that are at home each hour. As opposed to the Weekly Patterns data, which defines how people interact at POIs, this information is used to define the amount of time people are in contact with others from their household.

Census data provided by SafeGraph [26] is used to convert raw device counts into number of people using the visitor’s home CBG population and number of devices that reside in each CBG. The census data is also used to assign demographic information to each CBG, including median household size and median household income, which is also used in the analysis.

Six-digit North American Industry Classification System (NAICS) codes are extracted from SafeGraph Core Places dataset [22]. NAICS codes are used to classify each POI based on business type. This classification is used to help downselect locations used to build the contact network and identify trends and possible targeted policies to reduce transmission.

It is important to keep in mind that SafeGraph data does not capture every device and not all devices are linked to a home CBG. In the US, there are approximately 260.2 million smartphones users [27]. The data provided by SafeGraph represents approximately 6-8% of those devices, based on the data released between February and June of 2020. Devices that are out of service, not moving, lack a tracking app, or have opted out of location services are not included in the data. While the SafeGraph data includes a subset of the true population, the data includes the same places and individuals across time (to the extent possible) and the data has been analyzed to ensure it is representative of population demographics [28].

**Fig 2:**
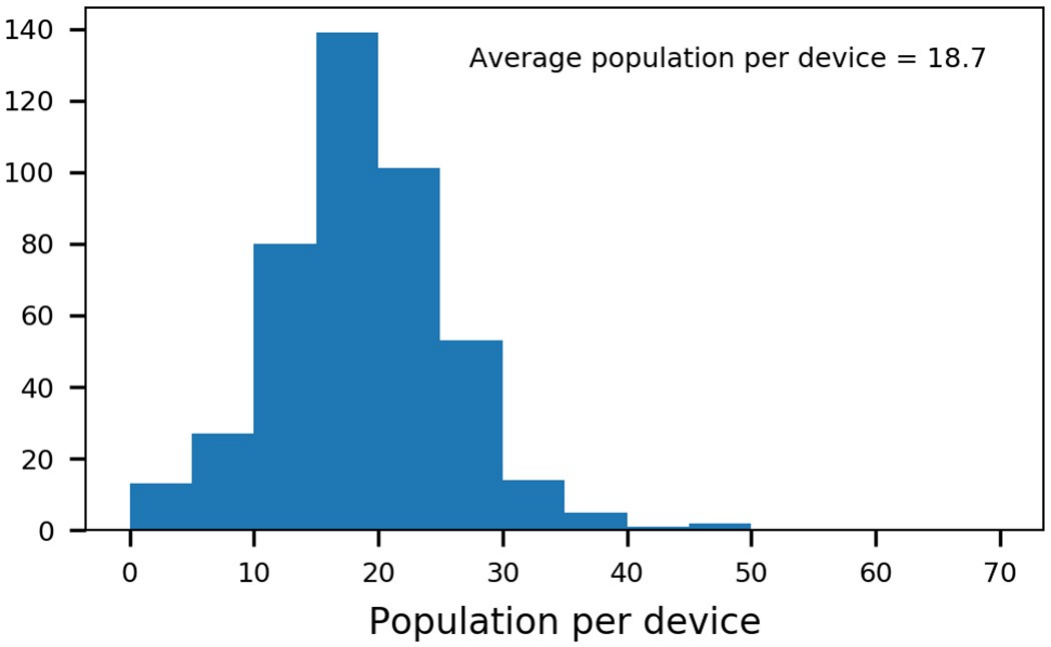
Distribution of population per device. Population per device was computed using each CBG in Bernalillo County, NM.

Device-registered visits are rescaled to reflect a larger percent of the general population by accounting for the number of people that each device represents. Fig 2 illustrates how the population per device varies across CBGs in Bernalillo County using data from February, 2020. Using the SafeGraph data that links device-registered visits to a home CBG, hourly device count at each POI is rescaled to estimate the number of visits. In Bernalillo County, 75% of visits are associated with a home CBG. Visits lacking an assigned CBG are scaled using the county average population per device (18.7 people per device).

### 2.2 Sampling locations and visitor arrival times

Sampling methods are often needed to extract patterns that represent population sizes smaller than the area’s true population. The sampling should reflect patterns observed in the data without requiring simulation of all individuals served by all commercial and public infrastructure the data reflects. Because we seek to explore the behavior of a specific intervention over a wide range of possible scenarios, the number of individuals represented in the contact network is limited to 10,000 to keep computation time reasonable. Larger networks could be used in future research.

The sampling method uses NAICS categories to group locations into business types. The number of locations per type varies considerably, for example with many restaurants and schools but few manufacturing plants and airports. If the modeled population reflects a fraction *s*_*p*_ of the actual population, and we want to capture the contact patterns that individuals in the larger population would create, there are two subsampling mechanisms available. First, individual locations can be culled so that the total number of locations of each type represents a fraction *s*_*p*_ of the original number of locations of that type. Second, the arrivals at each location can be thinned so that the number of individuals arriving represents the fraction *s*_*p*_ of the arrivals for the larger population. Subsampling locations preserves the occupation density at the retained locations and is therefore preferred. However some location types, such as an airport, have a single instance. Scaling the traffic at such locations to reflect only the modeled subset of the population can be thought of as reducing the size of the facility to match the modeled population.

**Fig 3:**
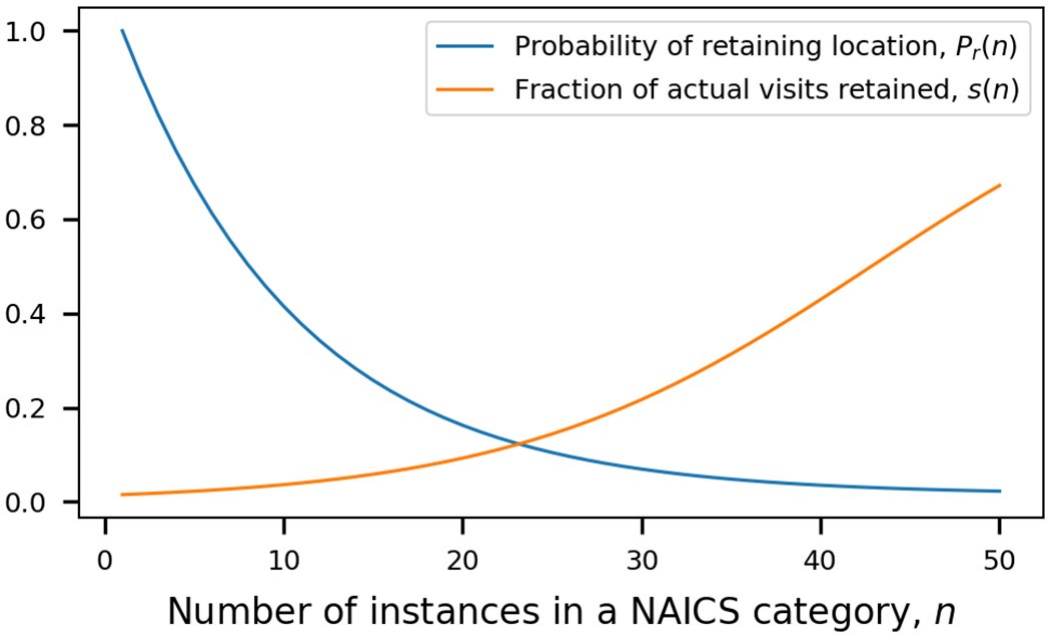
Scaling functions used to sample locations and the visits at each location. Scaling functions use N = 10 and *s*_*p*_ = 0.015.

Both sampling mechanisms are used to create a set of locations for each of the 6-digit NAICS codes represented in the data, and an associated sequence of arrival counts at each location in the set. For each NAICS code, the combined effect of the culling mechanism (to sample locations) and the thinning mechanism (to sample traffic at a location) produces a universal scaling factor equal to the fraction of the actual population the model population represents, as shown below.

$$P_{r}\left(n\right)=s_{p}+\left(1-s_{p}\right)e^{\frac{-(n-1)}{N}}$$ (1a)

$$s(n)=\frac{s_{p}}{P_{r}\left(n\right)}$$ (1b)

where *P*_*r*_(*n*) is the probability of retaining a specific location of type n, *s*(*n*) is the retained fraction of actual visits to locations of type n, *s*_*p*_ is the fraction of the region’s actual population represented in the model, *n* is the number of instances in locations of type n, and *N* is a global parameter that determines the number of instances associated with a transition from sampling traffic to sampling locations. The functions create a smooth transition in scaling mechanism as the number of instances increases, as shown in Fig 3.

Since contact opportunities come from concurrent occupancy, the timing of visits to a particular location are important. The fact that SafeGraph device counts represent a small fraction of visitors needs to be considered when constructing total arrivals for the scaled population. For a given location, suppose that recorded traffic represents a fraction *f*_*c*_ of actual traffic at the location. The fraction of actual traffic we wish to retain due to scaling considerations is *s*(*n*). If *s*(*n*) < *f*_*c*_ then there are more observations than required in the scaled data, and the fraction *l* = *s*(*n*)/*f*_*c*_ are retained. However, if *s*(*n*) ≥ *f*_*c*_ there are fewer observations than needed to represent total traffic at the scaled location. In this case, observed arrivals from other locations of the same type are combined with arrivals from the selected location to construct an arrival series that includes traffic from locations having a total traffic that is a factor of *l* as large as that of the original location. This method of construction seems likely to distribute the necessary new arrivals over time in a way that is characteristic of locations of that type, but without requiring development of a parametric model for arrival patterns for the type. Because *s*(*n*) (and therefore *l*) is large when *P*_*r*_(*n*) is small, when arrivals need to be borrowed from other locations only a few of those locations are selected to be included explicitly. The pool of unselected locations is therefore used as the source of supplementary arrivals.

**Fig 4:**
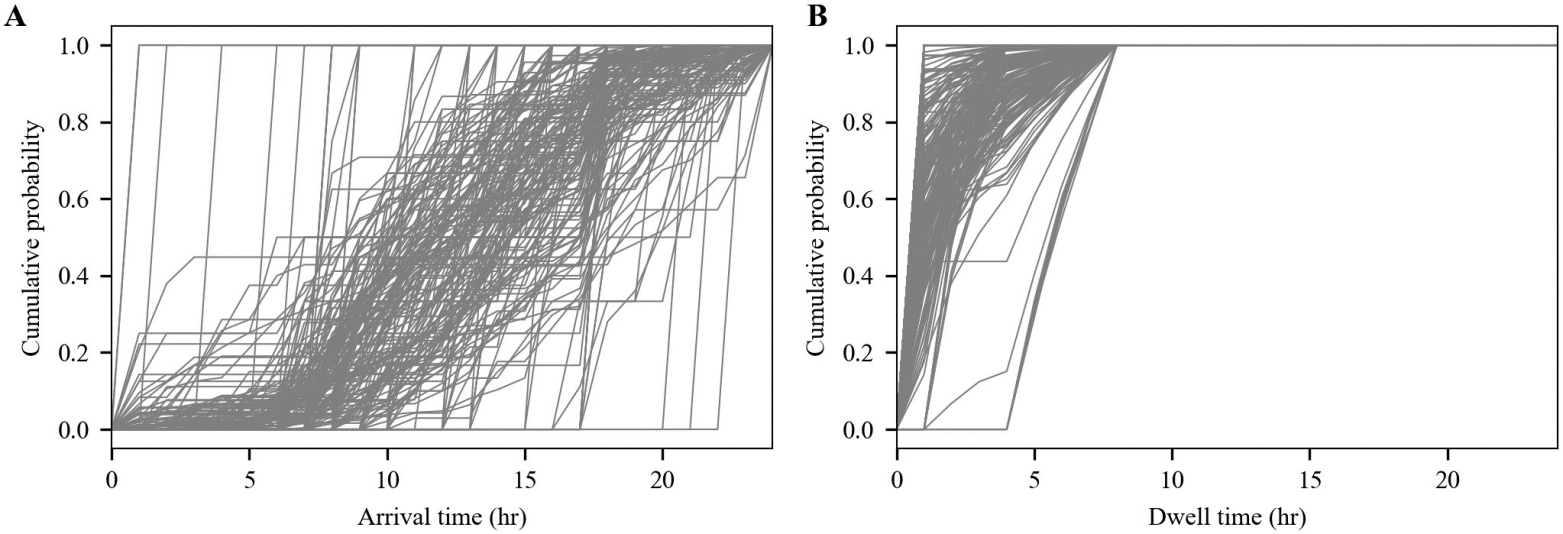
Arrival and dwell time distributions. Distributions include a subset of POIs in Bernalillo County. A: Arrival time. B: Dwell time.

The stochastic processes of selecting representative locations, and of selecting similar locations to supplement missing traffic result in arrival time distribution that are used to build the contact network. The arrival time distribution from a subset of POIs is shown in Fig 4. The sampling mechanisms are performed using 6-digit NAICS codes which, for example, distinguishes between POIs such as Cafeterias/Buffets (722514) from fast food (722211) from full service restaurants (722511). Additional demographic information could be included to ensure that characteristic traffic across different CBGs is preserved when combining locations [6].

### 2.3 Dwell time distributions

In addition to the arrival time distributions described above, dwell time distributions are needed to define how long individuals stay at a given place. Dwell times in the SafeGraph data are reported for each POI as the number of visits having durations within a fixed set of intervals (0-5 minutes, 5-20 minutes, 20-60 minutes 1-4 hours, and > 4 hours). The coarse resolution can make it difficult to replicate the median dwell time also reported by SafeGraph. For this reason, the binned dwell times were used to define an empirical distribution. Because dwell times in other domains are often seen to have a left-skewed distribution [29] (approximately modeled using exponential or gamma distributions) the logs of dwell times were assumed to be uniformly distributed between the (logs of) the interval boundaries. While the upper limit of dwell times is not available, one is required to define the uppermost interval. Some locations, such as hospitals, may have residence times exceeding one day. However, because the preponderance of locations are retail facilities, an upper limit of 8 hours was assumed. These assumptions lead to median simulated dwell time values that tend to agree with the reported medial dwell times, however there are discrepancies. First, the median simulated dwell times at some locations are much smaller than the reported medians. Some of these discrepancies occur at locations with little traffic. Focusing on locations with > 1000 people-hours of occupancy per week shows good agreement for median dwell times up to approximately 4 hours. Above 4 hours, reported median dwell times are systematically larger than the simulated values. This discrepancy is consistent with the upper dwell time limit being more than 8 hours, however that limit is not known. Note that if *s*(*n*) ≥ *f*_*c*_, then binned dwell times from the pool of unselected locations are used to create a composite binned dwell time for that location. If *s*(*n*) < *f*_*c*_, then selected locations use their assigned binned dwell times. Dwell time distributions from a subset of POIs is shown in Fig 4.

### 2.4 Household contacts

SafeGraph data does not include information on the arrival and dwell times at private residences. To represent household contacts in this analysis, the Census and Social Distance datasets were combined to group people into household clusters and determine the probability that people are at home on a hourly basis. These contacts are significant contributors to disease transmission, and so it is essential to consider them when assessing the power of limiting contacts in public settings to control disease spread. SafeGraph Social Distance data includes the number of devices estimated to be at home in each hour for each CBG, as well as the total number of devices residing in the CBG. The ratio estimates the fraction of the CBG population at home in each hour. Separately, Census data provide average household size for each CBG. We assume that household size follows a triangular distribution having the reported average, and generate a set of household sizes sufficient for the CBG’s model population. The hourly probability of being at home is then used to simulate the occupancy of each household during that hour.

### 2.5 Concurrent occupancy

The arrival and dwell time distributions for POIs, along with the probability that people are at home at any given hour are then used to determine if people are at the same location at the same time. These conditions create an opportunity for two or more people to interact. To ensure that people do not all show up at POIs on the hour, an arrival time is selected from a uniform distribution over the hour in which the visit is assigned. The departure time is then computed by drawing a dwell time from the location’s dwell time distribution. Household occupancy is established by sampling the number of residents at home in each hour, and randomly selecting that number from the set of household occupants. If multiple people are at a POI or household at the same time, their interaction is defined as the amount of time that their visits overlap. Contacts grow geometrically with the number of people concurrently at a location: 2 people create one contact, while 4 people create 6 distinct unordered pairs and so 6 contacts. Contact patterns among individuals at a POI will depend on many details of its design and the activities it supports. The tendency for contact opportunities to grow geometrically with occupancy is assumed to have some limit (for example people in a stadium do not interact with every other attendee); however, details about activity patterns within each POI are not available. Instead we define a threshold to limit the number of people that can come into contact at any given location. In this analysis, that value is set to 20 people for all locations. We have derived networks with thresholds of 10 and 30 to examine sensitivity to this threshold value. The distribution of contact densities is similar across threshold values. Additional modulation factors can also be added to modify the intensity of the contact based on roles (e.g., customer, employee), behavior (e.g., use of PPE), and the kinds of activities typically conducted at the location.

As people arrive at a POI, they are assigned to a node in the network based on the home CBG information provided by SafeGraph. This assignment is necessary because the aggregate data does not track mobility of individuals. To keep the analysis limited to a closed system, only CBGs within the region of interest are included as nodes. While visitors from outside the region of interest contribute to total interaction between visitors, they are not explicitly included in the model. The impact of out-of-region visitors is distributed among in-region nodes. This limitation could be resolved in future research.

Interaction terms due to concurrent occupancy at all locations are accumulated for all pairs of interacting individuals over the weekly period of record. These interaction terms are then normalized by the period of record so that they represent the fraction of time individuals spend in contact. This results in a unitless measure of interaction strength between each pair of nodes, *κ*_*i*,*j*_.

### 2.6 Contact intensity

The methods described above enable simulation of the amount of time two individuals spend together in specific circumstances, which is an important factor in estimating disease transmission probability. The nature of the interactions in those circumstances also matters. The differential transmission risk associated with each kind of public and private settings would ideally be used to weight the simulated interactions. Because this information is not readily available, we have explored the sensitivity of the contact networks to some alternative assumptions about variability of contact intensity with location type. Each of the 3-digit NAICS categories were assigned one of three levels of interaction intensity based on the category description and a subjective judgment about the tendency for individuals to be in close contact in locations of that kind. Three alternative functions were used to connect this assigned ranking to relative transmission risk: a unit value to all locations regardless of rank, a linear function of rank, and a quadratic function of rank (square of the rank). A unit risk was assigned to household activities in all cases.

## 3 Results

Contact networks were generated using several months of mobility data to study changes in network structure and the impact on disease transmission. The goal of this analysis is to better understand the types of businesses that have the highest levels of interaction and how network characteristics change over time. While the main focus of this paper is on network construction and characteristics, the contact networks can also be used in epidemiological models to investigate disease transmission and public policy. A brief example is included below and more details on the network-based SEIR modeling can be found in [23].

Contact networks were generated using SafeGraph data between February 10, 2020 and June 8, 2020, on a weekly basis using data from Bernalillo County, New Mexico. Bernalillo County has a population of 680,000 people, includes 435 CBGs and over 8000 POIs. Traffic at POIs is downscaled using the scaling parameters N = 10 and *s*_*p*_ = 0.015. This represents a population of 10,000 individuals. The data includes five weeks prior to state stay-at-home orders on March 11th. For each week, multiple realizations of the contact network were generated to investigate variability in the network characteristics.

For each network, we extracted the interaction strength between pairs of nodes, *κ*_*i*,*j*_. The interaction strength is a unitless value that measures the time per unit time that people spend in contact. The interaction strength for each node, *κ*_*i*_, an interaction strength for each POI, *κ*_*p*_, and a system average interaction strength, κ* are also included in the analysis below. All boxplots used to illustrate results include a box that extends from the first quartile (Q1) to the third quartile (Q3), whiskers that extend to 1.5 times the interquartile range (Q3-Q1), and a horizontal line at the median (Q2).

### 3.1 Network characteristics

**Fig 5:**
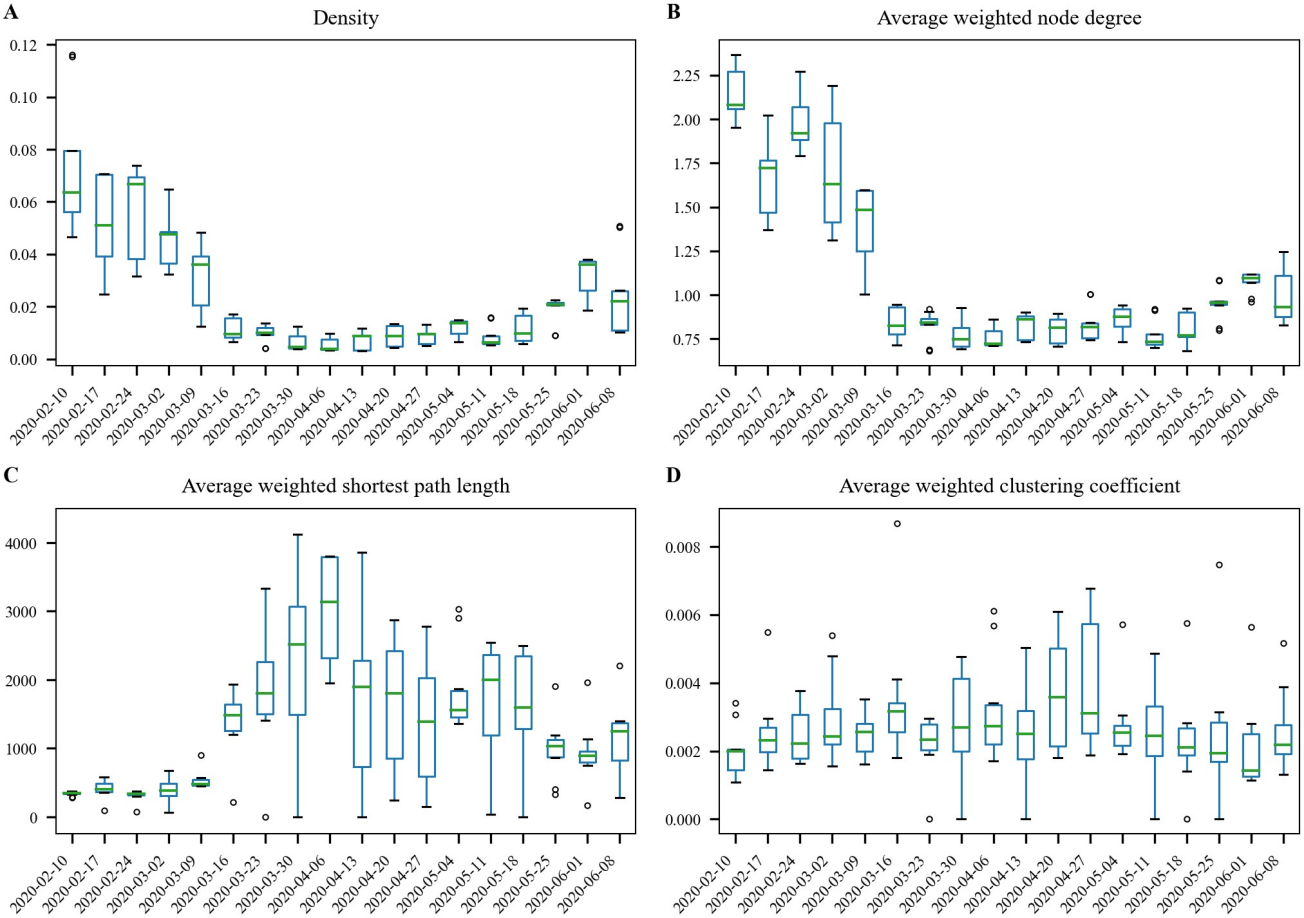
Network metrics between February and June. A: Density. B: Average node degree weighted by *κ*_*i*,*j*_. C: Average shortest path length weighted by 1/*κ*_*i*,*j*_. D: Clustering coefficient weighted by *κ*_*i*,*j*_. Each date includes 10 realizations of the network.

To understand how interactions and clustering of individuals change over time, several metrics were computed for each network, as shown in Fig 5. These metrics include network density, node degree, shortest path length, and clustering coefficient. The last three metrics were weighted using *κ*_*i*,*j*_. These metrics were selected to identify structural trends in the network as a function of time. All metrics were computed using NetworkX [30]. The different network realizations resulting from sampling locations and arrival events produce different networks, and therefore a range of values for each date. Additional information on weighted network metrics can be found in [31]. Calculation of the shortest path length and clustering coefficient are computationally expensive on large networks. For this reason, a subgraph using 10% of the nodes was randomly generated to compute these metrics. Results using different realizations of the subgraph show similar results.

- Network density is the number of edges in the network divided by the total possible number of edges. Density is 0 for a graph without edges and 1 for a complete graph. For networks that include loops and multiple edges between nodes, density can have a value greater than 1. For disease transmission, network density is a simple measure of potential social interaction.
- Node degree is the number of edges connected to each node. This metric can help differentiate people that interact with very few people with people that are potential super spreaders. Node degree is weighted by *κ*_*i*,*j*_ to identify the number of significant contacts in the network. The weighted node degree results in an interaction strength for each node, *κ*_*i*_. The average weighted node degree, κ*, is the average value across the network.
- Shortest path length computes the path of least resistance though the network from each pair of nodes. This metric is of particular interest when studying disease transmission, as people that are in contact for a longer time have an increased likelihood of spreading the disease. Since a large value of *κ*_*i*,*j*_ means that people are in contact for a longer amount of time, we use 1/*κ*_*i*,*j*_ to compute a weighted shortest path length. The average weighted shortest path length is the average value across the network.
- Clustering coefficient is a measure of the degree to which nodes in a graph cluster together. With respect to disease transmission, it is important to understand clustering, and paths between clusters, to predict how disease passes between groups of people. For example, if person A is in contact with persons B and C, it is also important to understand if persons B and C are in contact thereby forming a triangle. The clustering coefficient is weighted by *κ*_*i*,*j*_ to include the strength of these connections and potential clusters. The average weighted clustering coefficient is the average value across the network.

**Fig 6:**
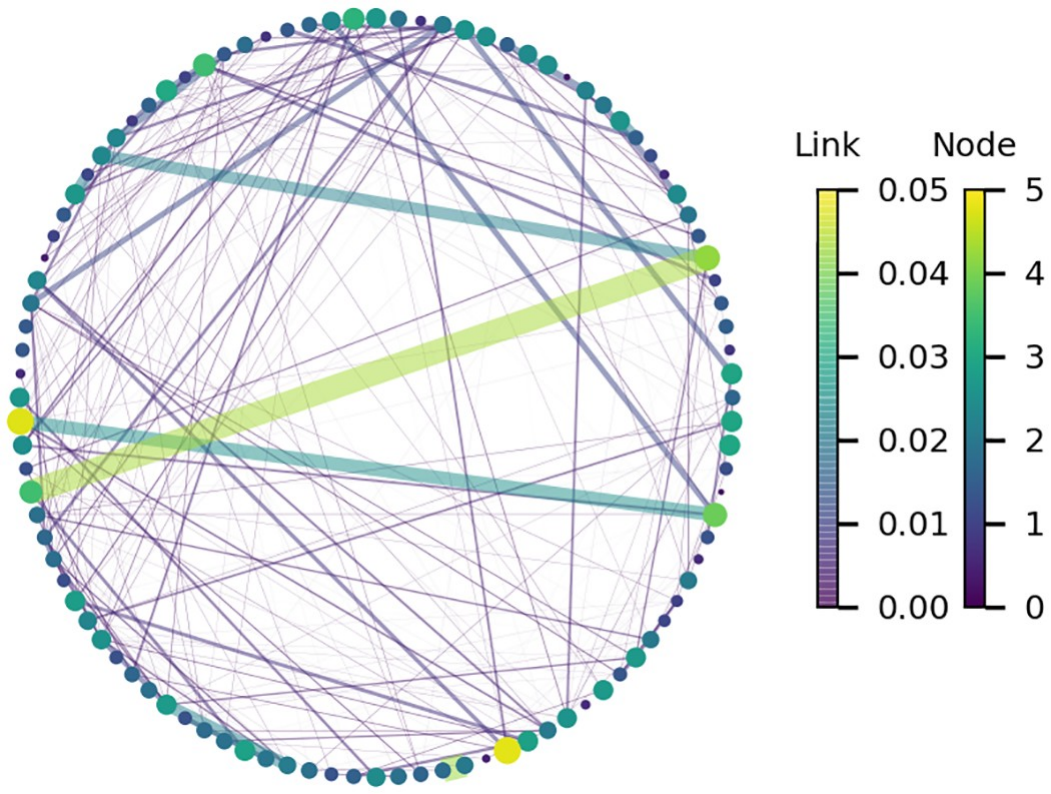
Subset of a contact network showing weighted node degree and interaction strength between nodes. Network generated using mobility data from February 10th. The node attribute is the weighted node degree, *κ*_*i*_, and the link attribute is interaction strength between nodes, *κ*_*ij*_. Line thickness and node diameter correspond to node degree and interaction strength.

The network metrics indicate that interactions clearly changed starting the week of March 16th. Network density and average weighted node degree decreased. This indicates that people are in contact with fewer people as a result of the policy change. The average weighted shortest path length shows a sharp increase and the average weighted clustering coefficient increased slightly in late April. This indicates that while people generally had fewer and shorter interactions (increasing the path of least resistance through the network), their social network had higher clustering. Since mobility data includes interactions that are outside peoples’ planned social circles (e.g., interactions at grocery stores), the increased clustering might indicate that people frequented a smaller set of locations as social distancing measures went into effect. Having a larger fraction of their interactions occur in households also increases this clustering. The network metrics show relatively consistent results between the week of March 16th and the week of May 25th (Memorial Day). After that time, the metrics revert slightly towards pre-lockdown levels. Fig 6 illustrates a subset of a contact network (1% of the network nodes) with weighted node degree, *κ*_*i*_, as the node attribute and interaction strength between nodes, *κ*_*i*,*j*_, as the link attribute. The small example illustrates strong links between a small number of nodes, and more frequent weaker links. Additional geographic trends could be explored in future research by visualizing these networks using the CBGs they represent.

**Fig 7:**
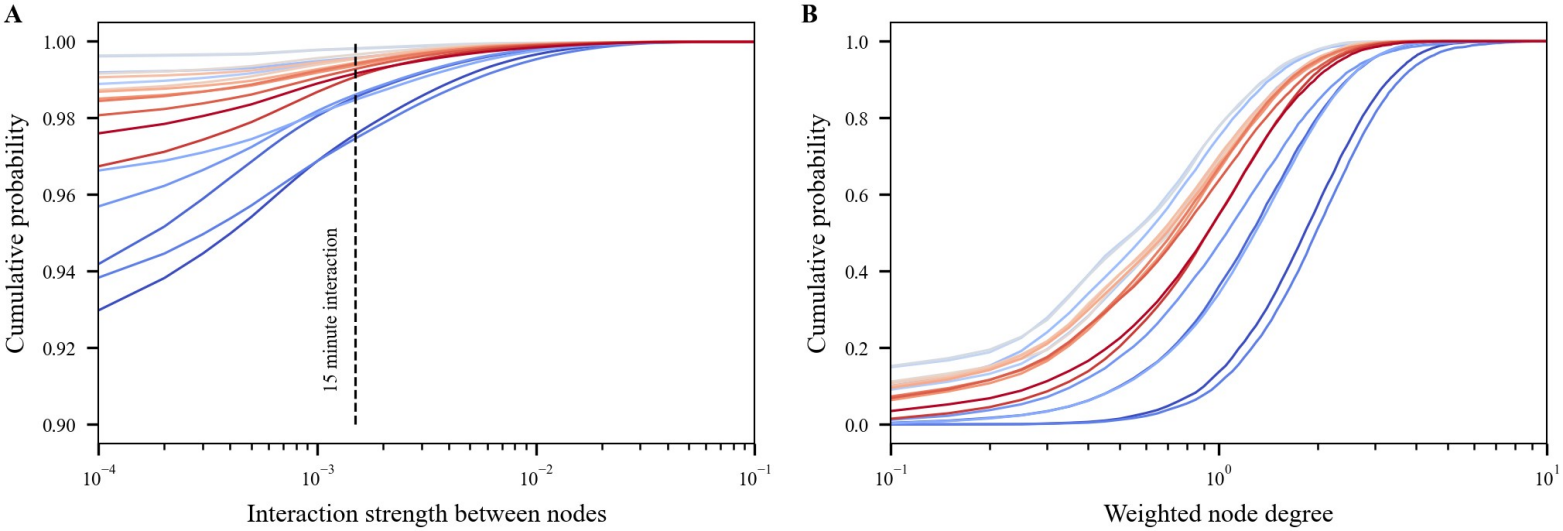
Interaction strength and weighted node degree distributions. The colorscale changes for each week, from February 10th (dark blue) to June 8th (dark red). A: Interaction strength between nodes, *κ*_*i*,*j*_. B: Weighted node degree, *κ*_*i*_.

**Fig 8:**
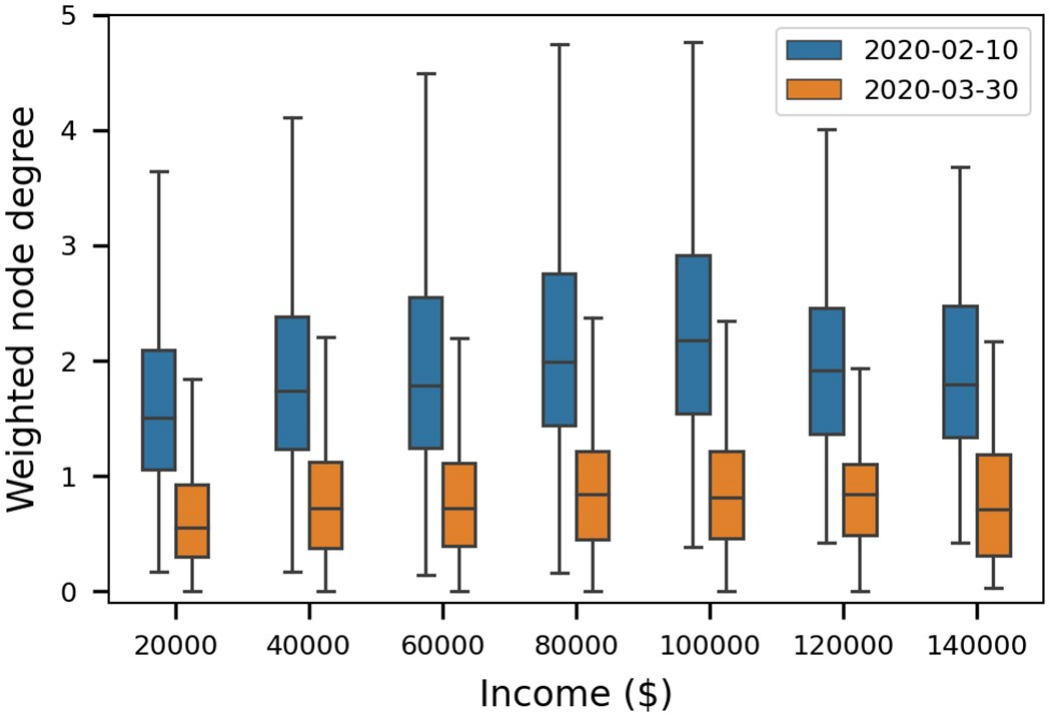
Weighted node degree by income bracket. Weighted node degree for February 10th and March 30th. Each income bracket ($20,000 increments) is labeled with the max value.

Fig 7 illustrates the cumulative probability of interaction between nodes, *κ*_*i*,*j*_, and weighted node degree, *κ*_*i*_, using networks generated for each week between February and June. Both metrics decrease substantially after stay-at-home orders. The interaction strength distribution shows that most contacts are less than the 15-minute threshold used by the CDC to define “close contact” [32]. While interaction strength is unitless, the dashed line in Fig 7 a designates that a person was in contact for 15 minutes over the course of a week (15/10080 = 0.0015). This indicates that over 97% of interactions are less than 15 minutes. The remaining contacts still represent an important subset of the population that can be included in contact tracing. For example, if the network density is 1%, then each person interacts with 100 people on average. If 1% are close contacts (with interactions lasting over 15 minutes), then this leaves 1 person which represents 68 people in a county the size of Bernalillo County. In the context of contact tracing, it is expected that a subset of people in close contact are recalled and will comply with testing. Because the individual network nodes are assigned to a CBG, metrics like weighed node degree can be further linked to demographics like median income. Fig 8 groups weighted node degree by income bracket. These results illustrate that while interaction across all income brackets decreased, the decrease was most pronounced in the $100,000 income bracket where interaction was highest before stay-at-home orders were put in place.

**Fig 9:**
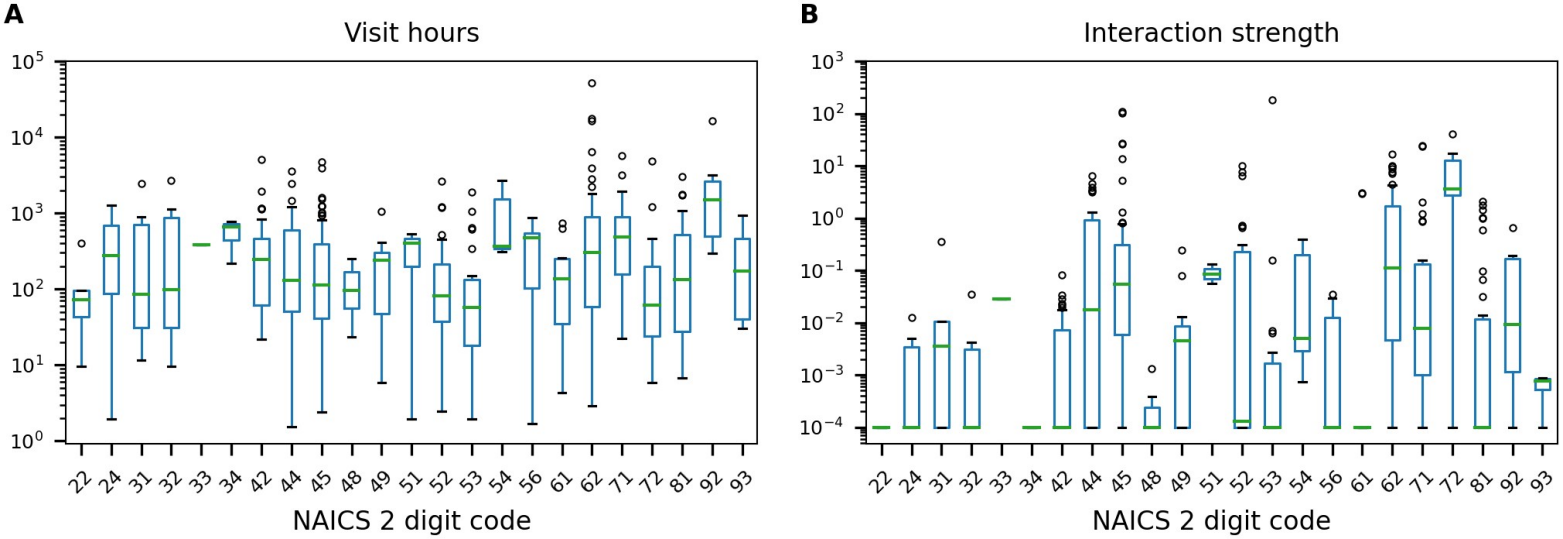
Traffic measured using visit-hours and interaction strength at POIs. POIs grouped by 2-digit NAICS code using data from March 30th. A: Visit-hours. B: Interaction strength, *κ*_*p*_.

In contrast to metrics that measure total traffic and visit-hours to POIs, interaction metrics take into account concurrent visits which are an important aspect to understanding potential transmission at POIs. Interaction strength at each POI, *κ*_*p*_, is the total interactions that occur at that site. Fig 9 shows both visit-hours and interaction strength for the POIs included in the Bernalillo County analysis for the week of March 30th (after stay-at-home orders were put into place). While the highest median visit-hours occurs at businesses related to public administration (2-digit NAICS code 92) the median interaction strength is highest at businesses related to accommodation and food services (2-digit NAICS code 72). Furthermore, several sectors generate very little interaction while still recording visitors. While these trends are not universal and are expected to change over time, the analysis illustrates important differences between visits and interactions. Better understanding of the types of interactions that occur at specific POIs can help target closures and mitigation strategies.

### 3.2 Impact on disease transmission

To use the contact networks in epidemiological models, the interaction terms must be converted to transmission probabilities. Contact networks derived using the weeks prior to mobility controls can be used to estimate the value of a single transmission probability parameter β*, based on the assumption that COVID-19 would have an initial doubling time of *T*_*d*_ days under those conditions. The population average interaction strength in a pre-lockdown network, κ*, is assumed to be associated with an unmitigated doubling time of *T*_*d*_. The β* value corresponding to this doubling time is then:

$$\begin{array}{c} \beta^{*}=\frac{ln(2)}{T_{d}\kappa^{*}} \end{array}$$ (2)

The values for κ* and β* for Bernalillo County using a doubling time of 2.5 days along with unit, linear, and quadratic contact intensity are shown in Table 1. While these values are reasonable with transmission probabilities ranging from 5 to 15%, the contact intensity with location type that was used to define the linear and quadratic levels were based on subjective judgment. Additional data and research is needed to better understand how contact intensity varies across locations. The β* values can also be used to convert the interaction strength between nodes, *κ*_*i*,*j*_, to transmission probabilities for use in disease models. Given the large number of very small interaction strength between certain individuals, the size of the network representation could be further reduced by eliminating these connections.

**Table 1:** Population averages for κ* and β* using different levels of contact intensity.

Contact intensity	κ*	β*
Unit	1.78	0.15
Linear	2.82	0.09
Quadratic	5.06	0.05

**Fig 10:**
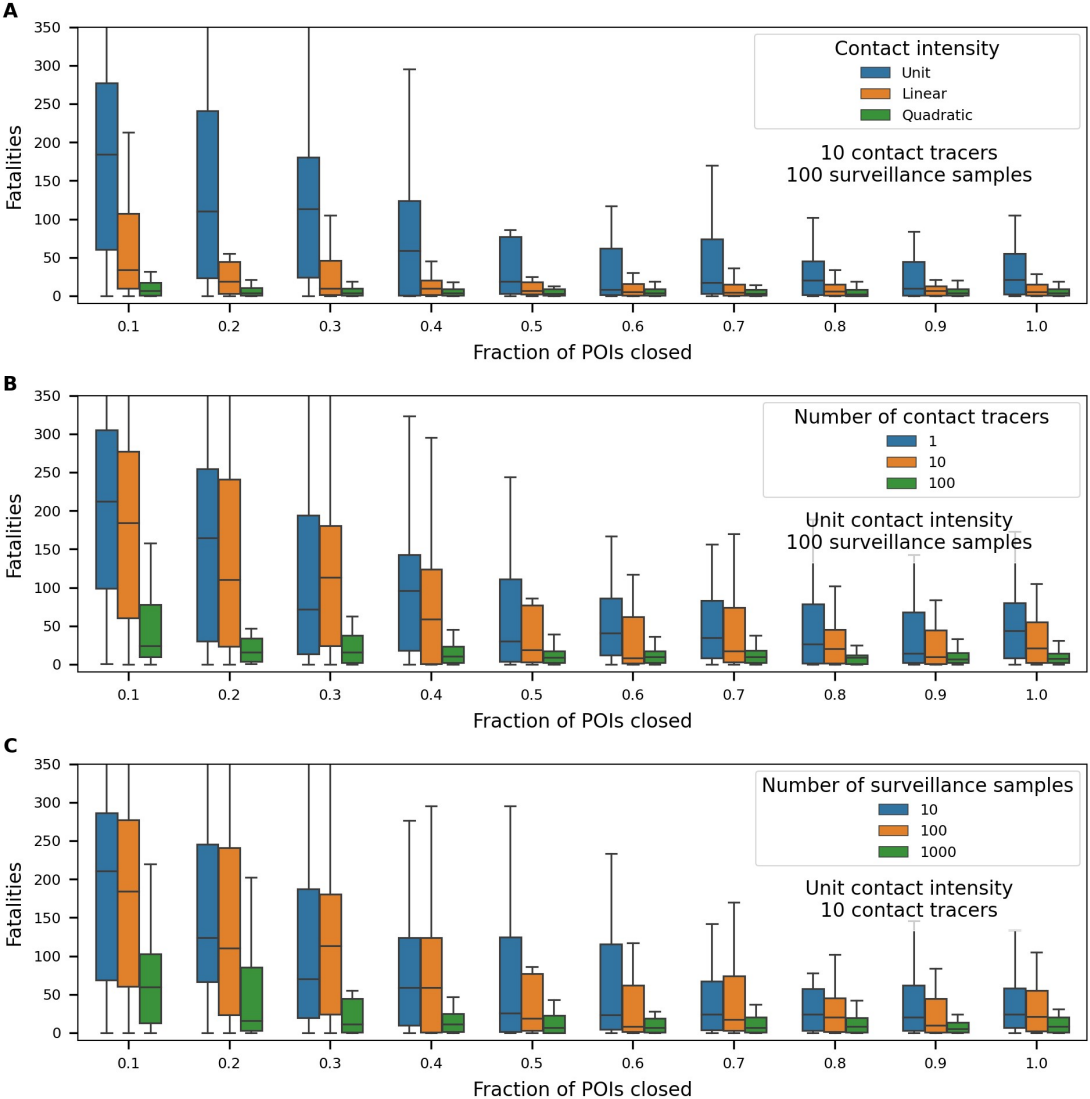
Epidemiological simulation results using the contact network. Impact of POI closure on fatalities in a one-year simulation of COVID-19 spread through 10,000 person contact networks. Subplots show the influence of A. contact intensity, B. number of contact tracers, and C. number of surveillance samples. Variables that are held constant in each subplot are noted below the legend.

The propensity for the interactions represented in the contact network to amplify or suppress disease propagation, and the comparative effectiveness of alternative control strategies to mitigate transmission potential, can be evaluated through epidemiological simulations. The Adaptive Recovery Model [23] was developed for this purpose. Fig 10 shows results that use the contact network construction. Modeled COVID-19 fatalities occurring over a one-year simulation period were explored using different levels of non-medical interventions, including the closure of POIs with high levels of interaction, staffing for contact tracing, and number of surveillance samples taken each day from randomly-chosen individuals. For reference, as of early February 2021, Bernalillo Country had reported 788 fatalities [33]. Based on the county’s population, this represents 11.5 people in the simulation results.

Precluding contacts at POIs prioritized by their interaction strength (*κ*_*p*_) is seen to suppress spread and lead to fewer fatalities, however transmission within households appears to dominate once contacts at the highest-ranked half of POIs are suppressed. Contact intensity has a large impact on results. As mentioned earlier, information on the nature of interaction in different circumstances is not readily available and requires further study. Quarantine strategies informed by contact tracing and surveillance sampling are also effective in controlling disease spread given adequate staffing, even when very few POIs are closed. In the simulations, the contact network structure also enables a fine-grained representation of the contact tracing process and the sampling process includes false negative rates. Future research will build on this analysis to help quantify the trade-off between different control strategies.

## 4 Discussion

Mobility data has tremendous potential to inform public health decision-making. Researchers have used such data to create indicators of activity changes in response to public health orders as well as to spontaneous aversion behavior. While observed reductions in public activity at an aggregate level can be correlated to later reductions in case counts, retrospective analyses of this kind are not ideal for guiding future decisions. Public health measures should be tailored to specific kinds of public activities. Those measures can be better informed if the activities at specific kinds of locations can be linked to possible epidemiological outcomes. For example, factors that increase community spread and transmission risk include crowded situations, close physical contact, enclosed poorly ventilated spaces, and duration or frequency of interaction.

We have analyzed mobility data to produce contact networks that represent a sample of individuals at the county scale. These sampled individuals act according to the mobility data, but because that data is anonymized they do not correspond to people in the real world. Contact networks encode the time that individuals spend together and the types of locations in which they interact. These networks can be used in different ways to guide pandemic containment policies. The network structures can be analyzed directly to obtain system-level insights about interaction patterns, for example the extent to which contacts occur in clusters, and the ease with which transmission can span the entire population. Such structural metrics summarize information about the patterns of relationships among the population, which cannot be derived from statistics of the original data. These patterns have clear implications for the rate and topology of spread.

The methods demonstrated in this paper can be automated to construct networks and perform the subsequent analysis. The spatial coverage and weekly updates of the SafeGraph data enable the analyses to be readily recreated for other regions and at other scales. The contact networks can be used directly in epidemiological simulations to test the effectiveness of policies focused on controlling activities or transmission potential at specific types of locations, as well as interventions focused on individuals with a high degree of person-to-person interaction.

## Supporting information

S1 File (PDF)

## References

[1] United States Centers for Disease Control and Prevention. Scientific Brief: SARS-CoV-2 and Potential Airborne Transmission; 2020. Available from: https://www.cdc.gov/coronavirus/2019-ncov/more/scientific-brief-sars-cov-2.html. 34009775

[2] Nishiura H, Oshitani H, Kobayashi T, Saito T, Sunagawa T, Matsui T, et al. Closed environments facilitate secondary transmission of coronavirus disease 2019 (COVID-19). medRxiv. 2020. 10.1101/2020.02.28.20029272

[3] Fisher KA, Tenforde MW, Feldstein LR, Lindsell CJ, Shapiro NI, Files DC, et al. Community and close contact exposures associated with COVID-19 among symptomatic adults≥ 18 years in 11 outpatient health care facilities—United States, July 2020. Morbidity and Mortality Weekly Report. 2020;69(36):1258. 10.15585/mmwr.mm6936a5 32915165 PMC7499837

[4] Courtemanche C, Garuccio J, Le A, Pinkston J, Yelowitz A. Strong Social Distancing Measures In The United States Reduced The COVID-19 Growth Rate: Study evaluates the impact of social distancing measures on the growth rate of confirmed COVID-19 cases across the United States. Health Affairs. 2020; p. 10–1377. 10.1377/hlthaff.2020.00608 32407171

[5] Cutler DM, Summers LH. The COVID-19 pandemic and the 16 trillion virus. Jama. 2020;324(15):1495–1496. 10.1001/jama.2020.19759 33044484 PMC7604733

[6] Chang S, Pierson E, Koh PW, Gerardin J, Redbird B, Grusky D, et al. Mobility network models of COVID-19 explain inequities and inform reopening. Nature. 2020; p. 1–6. 33171481 10.1038/s41586-020-2923-3

[7] Kumar N, Oke JB, Nahmias-Biran Bh. Activity-based contact network scaling and epidemic propagation in metropolitan areas. arXiv preprint: 200606039. 2020. 10.1038/s41598-021-01522-w PMC8608855 34811414

[8] Maheshwari P, Albert R. Network model and analysis of the spread of COVID-19 with social distancing. Applied network science. 2020;5(1):1–13. 10.1007/s41109-020-00344-5 33392389 PMC7770744

[9] Muller SA, Balmer M, Neumann A, Nagel K. Mobility traces and spreading of COVID-19. medRxiv preprint. 2020.

[10] Rechtin M, Feldman V, Klare S, Riddle N, Sharma R. Modeling and Simulation of COVID-19 Pandemic for Cincinnati Tri-State Area. arXiv preprint: 200606021. 2020.

[11] Schlosser F, Maier BF, Jack O, Hinrichs D, Zachariae A, Brockmann D. COVID-19 lockdown induces disease-mitigating structural changes in mobility networks. Proceedings of the National Academy of Sciences. 2020;117(52):32883–32890. 10.1073/pnas.2012326117 33273120 PMC7776901

[12] Soures N, Chambers D, Carmichael Z, Daram A, Shah DP, Clark K, et al. SIRNet: Understanding social distancing measures with hybrid neural network model for COVID-19 infectious spread. arXiv preprint: 200410376. 2020.

[13] Yi H, Ng ST, Farwin A, Pei Ting Low A, Chang CM, Lim J. Health equity considerations in COVID-19: geospatial network analysis of the COVID-19 outbreak in the migrant population in Singapore. Journal of Travel Medicine. 2020. 10.1093/jtm/taaa159 PMC7499763 32894286

[14] SafeGraph. SafeGraph COVID-19 Data Consortium; 2020. Available from: https://www.safegraph.com/covid-19-data-consortium.

[15] Google. Google COVID-19 Community Mobility Reports; 2020. Available from: https://www.google.com/covid19/mobility.

[16] Dave D, Friedson AI, McNichols D, Sabia JJ. The Contagion Externality of a Superspreading Event: The Sturgis Motorcycle Rally and COVID-19. Institute of Labor Economics; 2020. IZA DP No. 13670. 10.1002/soej.12475 PMC7753804 33362303

[17] Lasry A, Kidder D, Hast M, Poovey J, Sunshine G, Winglee K, et al. Timing of community mitigation and changes in reported COVID-19 and community mobility–four US metropolitan areas, February 26–April 1, 2020. Morbidity and Mortality Weekly Report. 2020;69(15):451–457. 10.15585/mmwr.mm6915e2 32298245 PMC7755061

[18] McMinn S, Talbot R. Mobile Phone Data Show More Americans Are Leaving Their Homes, Despite Orders; 2020. Available from: https://www.npr.org/2020/05/01/849161820/mobile-phone-data-show-more-americans-are-leaving-their-homes-despite-orders.

[19] Pan Y, Darzi A, Kabiri A, Zhao G, Luo W, Xiong C, et al. Quantifying human mobility behaviour changes during the COVID-19 outbreak in the United States. Scientific Reports. 2020;10(1):1–9. 10.1038/s41598-020-77751-2 33244071 PMC7691347

[20] Weill JA, Stigler M, Deschenes O, Springborn MR. Social distancing responses to COVID-19 emergency declarations strongly differentiated by income. Proceedings of the National Academy of Sciences. 2020;117(33):19658–19660. 10.1073/pnas.2009412117 32727905 PMC7443940

[21] Ma KC, Lipsitch M. Big data and simple models used to track the spread of COVID-19 in cities; 2020. Available from: https://www.nature.com/articles/d41586-020-02964-4. 10.1038/d41586-020-02964-4 33173216

[22] SafeGraph. Core Places; 2021. Available from: https://docs.safegraph.com/v4.0/docs/section-core-places.

[23] Beyeler W, Acquesta E, Klise K, Makvandi M, Finley P. Adaptive Recovery Model: Designing Systems for Testing, Tracing, and Vaccination to Support COVID-19 Recovery Planning. Sandia National Laboratories; 2020.

[24] SafeGraph. Weekly Patterns (v2); 2021. Available from: https://docs.safegraph.com/v4.0/docs/weekly-patterns.

[25] SafeGraph. Social Distancing Metrics; 2021. Available from: https://docs.safegraph.com/v4.0/docs/social-distancing-metrics.

[26] SafeGraph. Census Block Group Data; 2021. Available from: https://docs.safegraph.com/docs/open-census-data.

[27] Newzoo. Global Mobile Market Report. Newzoo; 2019.

[28] Squire, Ryan Fox. What about bias in the SafeGraph dataset?; 2019. Available from: https://www.safegraph.com/blog/what-about-bias-in-the-safegraph-dataset.

[29] Hogg RV, McKean JW, Craig AT. Introduction to Mathematical Statistics 8th ed. Pearson; 2019.

[30] Hagberg AA, Schult DA, Swart PJ. Exploring network structure, dynamics, and function using NetworkX. In: G Varoquaux JM T Vaught, editor. 7th Python in Science Conference (SciPy2008); 2008. p. 11–16.

[31] Bassett DS, Bullmore ET. Small-world brain networks revisited. The Neuroscientist. 2017;23(5):499–516. 10.1177/1073858416667720 27655008 PMC5603984

[32] United States Centers for Disease Control and Prevention. Contact Tracing for COVID-19; 2020. Available from: https://www.cdc.gov/coronavirus/2019-ncov/php/contact-tracing/contact-tracing-plan/contact-tracing.html.

[33] The New York Times. Coronavirus in the U.S.: Latest Map and Case Count; 2021. Available from: https://www.nytimes.com/interactive/2020/us/coronavirus-us-cases.html.

