# Supplementary material for: Analysis of mobility data to build contact networks for COVID-19
Source: PLoS One. 2021 Apr 15;16(4):e0249726. doi: 10.1371/journal.pone.0249726 (PMC8049304; doi:10.1371/journal.pone.0249726)
Supplement: S1 File — (PDF) [file pone.0249726.s001.pdf]

SANDIA REPORT

SAND2020-11014
Printed

Sandia National Laboratories

Adaptive Recovery Model: Designing Systems for Testing, Tracing, and Vaccination to Support COVID-19 Recovery Planning

Walt Beyeler, Erin Acquesta, Katherine Klise, Monear Makvandi, Patrick Finley

Prepared by
Sandia National Laboratories
Albuquerque, New Mexico 87185
Livermore, California 94550

Issued by Sandia National Laboratories, operated for the United States Department of Energy by National Technology & Engineering Solutions of Sandia, LLC.

NOTICE: This report was prepared as an account of work sponsored by an agency of the United States Government. Neither the United States Government, nor any agency thereof, nor any of their employees, nor any of their contractors, subcontractors, or their employees, make any warranty, express or implied, or assume any legal liability or responsibility for the accuracy, completeness, or usefulness of any information, apparatus, product, or process disclosed, or represent that its use would not infringe privately owned rights. Reference herein to any specific commercial product, process, or service by trade name, trademark, manufacturer, or otherwise, does not necessarily constitute or imply its endorsement, recommendation, or favoring by the United States Government, any agency thereof, or any of their contractors or subcontractors. The views and opinions expressed herein do not necessarily state or reflect those of the United States Government, any agency thereof, or any of their contractors.

Printed in the United States of America. This report has been reproduced directly from the best available copy.

Available to DOE and DOE contractors from

U.S. Department of Energy
Office of Scientific and Technical Information
P.O. Box 62
Oak Ridge, TN 37831

Telephone: (865) 576-8401
Facsimile: (865) 576-5728
E-Mail: reports@osti.gov
Online ordering: http://www.osti.gov/scitech

Available to the public from

U.S. Department of Commerce
National Technical Information Service
5301 Shawnee Road
Alexandria, VA 22312

Telephone: (800) 553-6847
Facsimile: (703) 605-6900
E-Mail: orders@ntis.gov
Online order: https://classic.ntis.gov/help/order-methods

ABSTRACT

This report documents a new approach to designing disease control policies that allocate scarce testing, contact tracing, and vaccination resources to better control community transmission of COVID19 or similar diseases. The Adaptive Recovery Model (ARM) combines a deterministic compartmental disease model with a stochastic network disease propagation model to enable us to simulate COVID-19 community spread through the lens of two complementary modeling motifs. ARM contact networks are derived from cell-phone location data that have been anonymized and interpreted as individual arrivals to specific public locations. Modeling disease spread over these networks allows us to identify locations within communities conducive to rapid disease spread. ARM applies this model- and data-derived abstractions of community transmission to evaluate the effectiveness of disease control measures including targeted social distancing, contact tracing, testing and vaccination. The architecture of ARM provides a unique capacity to help decision makers understand how best to deploy scarce testing, tracing and vaccination resources to minimize disease-spread potential in a community.

This document details the novel mathematical formulations underlying ARM, presents a dynamical stability analysis of the deterministic model components, a sensitivity analysis of control parameters and network structure, and summarizes a process for deriving contact networks from cell-phone location data.

An example use case steps through applying ARM to evaluate three targeted social distancing policies using Bernalillo County, New Mexico as an exemplar test locale. This step-by-step analysis demonstrates how ARM can be used to measure the relative performance of competing public health policies. Initial scenario tests of ARM shows that ARM's design focus on resource utilization rather than simple incidence prediction can provide decision makers with additional quantitative guidance for managing ongoing public health emergencies and planning future responses.

ACKNOWLEDGMENT

The authors would like to thank Sean DeRosa for direction and support.

Research was supported by the DOE Office of Science through the National Virtual Biotechnology Laboratory, a consortium of DOE national laboratories focused on response to COVID-19, with funding provided by the Coronavirus CARES Act.

Disclaimer Unless otherwise indicated, this information has been authored by an employee or employees of National Technology and Engineering Solutions of Sandia, LLC, operator of Sandia National Laboratories with the U.S. Department of Energy. The U.S. Government has rights to use, reproduce, and distribute this information. The public may copy and use this information without charge, provided that this Notice and any statement of authorship are reproduced on all copies. While every effort has been made to produce valid data, by using this data, User acknowledges that neither the Government nor operating contractors of the above national laboratories makes any warranty, express or implied, of either the accuracy or completeness of this information or assumes any liability or responsibility for the use of this information. Additionally, this information is provided solely for research purposes and is not provided for purposes of offering medical advice. Accordingly, the U.S. Government and operating contractors of the above national laboratories are not to be liable to any user for any loss or damage, whether in contract, tort (including negligence), breach of statutory duty, or otherwise, even if foreseeable, arising under or in connection with use of or reliance on the content displayed in this report.

CONTENTS

1. Overview **9**

2. Status of Resource Estimates for Contact Tracing and Testing **11**

3. Adaptive Recovery Model Formulation **14**
3.1. Deterministic Model 15
3.1.1. Vaccine Distribution 18
3.1.2. Contact Tracing 19
3.1.3. Random Testing 21
3.2. Stochastic Network Model 21

4. Dynamical System Analysis **26**

5. Network Structure Sensitivity **31**

6. Contact Networks using Foot Traffic Data **37**

7. Applications and Future Directions **39**
7.1. Overview and Approach 39
7.2. Process Steps 40
7.3. Evaluation of Controls 42
7.4. Summary and Future Directions 45

8. Conclusions **48**

References **49**

LIST OF FIGURES

Figure 3-1. Compartment Model States and Transitions 15
Figure 3-2. Deterministic and Stochastic Network Model Comparison 25

Figure 4-1. State Simulations for a Controlled Outbreak 28
Figure 4-2. State Simulations for an Un-controlled Outbreak 29

Figure 5-1. Deterministic Model Sensitivity Study, Full 33
Figure 5-2. Deterministic Model Sensitivity Study, Subset 34
Figure 5-3. Stochastic Model Sensitivity Study, Subset 35
Figure 5-4. Stochastic Model Sensitivity Study, Subset, Uniform Degree 35
Figure 5-5. Stochastic Model Sensitivity Study, Subset, BA Filter 36
Figure 5-6. Stochastic Model Sensitivity Study, Subset, BA Filter with κ Targeting 36

Figure 7-1. Average Contact Density vs. Time 41
Figure 7-2. Modeled Fatalities after One Year assuming each Week's Contact Patterns Persisted 42
Figure 7-3. Distributions of Modeled Fatalities after One Year for Various Combinations of Control Parameter Values: No Business Closures 44
Figure 7-4. DIstributions of Modeled Fatalities after One Year for Various Combinations of Control Parameter Values: Closure of Businesses in 8 High-contact Sectors 45
Figure 7-5. Distributions of Modeled Fatalities after One Year for Various Combinations of Control Parameter Values: Closure of Businesses in "Non-Essential" Sectors 46

LIST OF TABLES

Table 3-1. Compartment Model Population State Definitions 14
Table 3-2. Compartmental Model Disease Related Parameter Definitions 17
Table 3-3. Compartmental Model Mitigation Related Parameter Definitions 18

Table 4-1. Numerical Values for Deterministic Simulation Parameters 27

Table 5-1. Epidemiological Parameter Variance for Sensitivity Study 32
Table 5-2. Control Parameter Variance for Sensitivity Study 32

Table 7-1. Epidemiological Parameter Variance for Sensitivity Study 43

NOMENCLATURE

Abbreviation	Definition
CBG	Census Block Group
COVID-19	Corona Virus Disease 2019
DOE	Department of Energy
GUI	Graphical User Interface
MERS	Middle-East Respiratory Syndrome
NMI	Non-medical Intervention
ODE	Ordinary Differential Equation
POI	Point of Interest
PPE	Personal protective Equipment
SARS	Severe Acute Respiratory Syndrome
SEIR	Susceptible-Exposed-Infected-Recovered epidemiology model

1. OVERVIEW

This report documents research on a new analytical method, called the Adaptive Recovery Model (ARM), to better control outbreaks of COVID-19 in the United States. Social-distancing policies have been successful at lowering the number of new cases in many states, indicating that general reduction in the number of contacts among all members of the population is effective in lowering the number of individuals contracting the disease and those who die from it. This general reduction in contacts entails suspension of many kinds of social and economic activity. As those social distancing policies were relaxed to enable economic recovery, new outbreaks of COVID-19 spread through the population, precipitating additional closures and restrictions, and requirements to wear masks in many places. Containing COVID-19 spread involves finding an acceptable combination of non-medical countermeasures pending availability of a vaccine. Once a vaccine becomes available, distributing the initially limited supplies will raise additional questions about control strategy design.

Methods that allow analysts and decision-makers to understand the interplay of the control measures available to them with the disease dynamics can be a great aid in discovering effective and robust designs. The goal of this project is to create a sound technical basis for deriving practical requirements for tracing and surveillance systems, and distribution of vaccines when they become available, which would allow social distancing measures and business constraints to be narrowly targeted. While important modeling components are available for examining disease spread and containment via tracing and surveillance, available models are predicated on unrealistic idealizations of contact patterns among individuals or are anchored in highly specialized networks that can't be generalized. Fine-grained foot-traffic mobility data allow for representative contact networks to be synthesized, and the potential effects of policy changes focused on specific kinds of locations or activities to be estimated.

The work summarized here has completed the technical foundation for pursuing the urgent goal of containment design. Containment system components consist of testing, contact tracing, closures and restrictions on business and other public activities, measures to foster use of personal protective equipment like masks (PPE),and vaccination. We have analyzed the dynamics of the coupled disease transmission with the control system. The coupled disease/control dynamics have been implemented both as a deterministic compartment model and as a stochastic network model. The deterministic model has been analyzed to provide insights into the relationship between model parameters and controllability. Contact networks for the stochastic model can be specified by analysis of mobility data, adjusted to reflect potential effects of policies on behavior and transmission, and evolving community behaviors over the course of a pandemic.

Current work on designing tracing and testing systems in the context of the COVID-19 pandemic is reviewed in Section 2. A modeling framework that focuses on the critical system processes, that incorporates both aggregated and explicit models for inter-personal contacts, is motivated and described in Section 3. Section 4 provides a discussion on equilibria assessments under alternative model formulations along with the determinant of endpoints for the given mechanistic structure. The stochastic network model is used to explore the effects of contact network structure in Section 5. Section 6 describes methods to estimating network structure from near-real-time cell phone tracking data analyzed to estimate density and

mobility. Section 7 discusses applications and future directions.

This work has created key components of a method for designing contact tracing and surveillance strategies, as well as deployment priorities for anticipated vaccines, having the capacity to control the increased outbreak risk created by relaxed social distancing, and to inform the design and operation of these systems using cell phone data on mobility and density. The method can be applied using location-specific information to develop sampling system designs tailored to a region's activity patterns and initial disease prevalence.

2. STATUS OF RESOURCE ESTIMATES FOR CONTACT TRACING AND TESTING

Rapid and widely available testing is a recognized component to any disease control strategy for COVID-19. Transmission speed, the global scale of the pandemic, and difficulties and delays in expanding testing capacity in the United States have combined to create constraints on the number of tests for COVID-19 infection that can currently be processed. As a result, the infectious status of much of the population is unknown. This ignorance leaves wholesale reductions in contacts among individuals, such as stay-at-home directives, as the only available public health response for controlling the outbreak. In contrast, case control strategies based on contact tracing and surveillance create visibility into disease prevalence in the population. When effective, they justify the default assumption that general members of the population are not infected, and so enable less constrained social and economic interactions.

Recent estimates on the amount of testing and contact tracing that would be required to achieve this transition from blanket social distancing to decreased/targeted social distancing vary widely, and are based on different assumptions and techniques. On March 28th, Gottlieb et al. [4] published an early estimate for the testing needs to support contact-based control. Based on the 2017-2018 flu season in the US and by translating Korea's COVID-19 experience to the US on a per-capita basis, they estimate a rate of 750,000 tests per week to be required. Siddarth and Wyle [18] discuss the fundamental challenge involved in moving to contact-based control: cases need to be found and effectively isolated more quickly than new uncontrolled cases are generated. They use a simple dynamical analysis to derive estimated testing requirements at the national level as large as millions per week, depending on efficiency of targeting and test accuracy. Their analysis illustrates the critical performance differences between appropriately sized and undersized systems, and how pragmatic factors such as accuracy and targeting efficiency can strongly influence sufficiency criteria.

Sanche et al. [17] estimate parameters of an epidemiological model based on case data from Wuhan province, and use that model to study the implications for control. Their formulation critically includes the possibility of asymptomatic transmissions. Parametric tradeoffs suggest that both accurate detection and effective quarantine of the infected population would be required to force the effective reproduction number below 1. Ngonghala et al. [14] developed a model involving both disease states and the control status of individuals in various disease states. Their formulation includes asymptomatic transmission, quarantining of exposed and infected individuals, contact tracing, and personal protective equipment (PPE) used in public and hospital settings. They use this model to evaluate the power of various non-medical interventions (NMIs) to suppress outbreak. They find that masks and social distancing continue to be needed; and that quarantines and contact tracing are marginally effective in reducing peaks in case counts.

McGee [11] released an open source software package that models epidemic dynamics with network-structured populations. The model includes testing, contact tracing, and social distancing using both deterministic ordinary differential equations (ODEs) and stochastic network-based representation of the system. As in the Ngonghala model, some disease states are factored into controlled and uncontrolled parts of the population, analyzing the processes used to identify and quarantine potentially infected people.

The model uses parameters for the general SEIR formulation derived for COVID-19, however it does not distinguish asymptomatic from symptomatic infected states, does not explicitly update contacts from the infected population, and does not facilitate prioritization of contact tracing. Given the use of common disease states in the ODE and network representations, they converge in the case of large populations. However the network creates a mechanism for exploring the effects of heterogeneous contact patterns, and for examining control strategies that exploit them (e.g. Glass et al, [3]).

Hellewell et al. [5] employ a stochastic branching model to assess tracing requirements for containment. In contrast to aggregate state models of population disease considered (e.g. [17, 14]) this formulation captures the effects of individual variability in contact network complexity. They define the population using a single set of distributions for relevant parameters; however, the approach is amenable to defining distinct population groups. They assume that asymptomatic cases are never isolated, and that traced individuals are isolated only when they become symptomatic.

Peak et al. [15] investigate the power of non-pharmaceutical controls including contact tracing, quarantine, and encouragement to self-report based on symptom monitoring. They study the effectiveness of different degrees of control in suppressing outbreak based on epidemiological parameters including the basic reproduction number R_0, potential for non-symptomatic transmission, and the relative timing of symptom onset and infectiousness. Although their work predates COVID-19, they consider parameter ranges that characterize a number of infectious diseases (SARS, Influenza A, Pertussis, Ebola, Hepatitis A, Smallpox, MERS). Diseases characterized by pre-symptomatic and asymptomatic transmission were found to be difficult to control using measures short of quarantine.

Aleta et al. [1] study requirements for contact tracing systems to suppress a second-wave outbreak following relaxation of social distancing controls. They configure an agent-based model using anonymized data collected from mobile devices in Boston from October 2016 to March 2017, combined with demographic data used to define household composition. They use the model to study resurgence under different scenarios for timing of relaxation of social distancing measures and performance of the contact tracing program. They find that locating 50% of symptomatic cases, and quarantining 40% of the contacts of those cases along with their households, protects against overwhelming loads on hospitals.

Firth et al. [2] also use mobile device data to generate possible contact networks in order to study the performance of different contact tracing strategies. Like Aleta et al. [1] they find that some control of contacts-of-contacts can be much more effective than only controlling first-order contacts. Their assessment uses a detailed data set from Haslemere UK collected in 2017/2018 specifically to study disease transmission through social networks. They find network structure to be an important influence on the estimated number of quarantined cases, however the fraction of the model population ultimately infected was similar for the data-derived network and a set of null networks preserving only certain statistics of the data-derived network. The authors consider the effect of physical distancing (modeled by consolidation of contacts around more-frequently contacted individuals while preserving total contact rate) and find little reduction in the total number of cases either along or in conjunction with contact tracing.

Kwok et al. [8] review seven epidemiological models of SARS and MERS transmission used to assess contact tracing and follow-up control measures. The reviewed models included both population compartment models and individual-based models. One model distinguishes student groups from the general population, the remainder assume homogeneous mixing. The review summarizes whether, and in what way, various aspects of tracing and control (testing, quarantine, contact tracing) are represented; including whether or not implementing delays and errors are considered. The authors call for consideration

of capacity limits and other logistical factors in modeling containment mechanisms.

Engineering systems that can identify and contain individual cases and case clusters with high reliability requires consideration of several factors and their interactions. The fundamental requirement is to detect and contain new cases faster than they can be produced through new transmissions. Designing control solutions entails considering diverse kinds of processes: diseases spread, social interactions, information acquisition, and administration of control measures. Existing studies have examined important parts of the problem (significance of transmission speed, asymptomatic transmission, the importance of outstanding case counts in determining the viability of contract tracing regimes, error rates of tests) but we have found none that integrates all of the relevant factors in a way that couples disease dynamics with operations of the control systems so that decision-makers can understand the interplay on the scale of their system. By including logistical constraints that may limit system performance (e.g. latency in getting test results, time to reach contacts and their compliance with requests to quarantine), along with resolution on occupational locations and events, we will provide the ability to assess the value of focused testing or contact control mechanisms (e.g., targeted closure orders).

3. ADAPTIVE RECOVERY MODEL FORMULATION

Available estimates of the prospects of containing COVID-19 through contact tracing and surveillance demonstrate the need to consider the interplay of several factors when designing the transition to contact-based containment (See Chapter 2). Inspired by the formulation provided by Ngonghala et al. [14] and the software framework from McGee [11], we developed the Adaptive Recovery Model (ARM) which combines current understanding of the disease states and control processes essential to the problem. We are aware of no existing formalism that combines them to address the general system design problem at a scale that is relevant to municipal authorities that need to equip and manage public health responses.

Adapting McGee [11], we define dual formulations of a component model that includes the epidemiological consequences of contact tracing and testing as well as a single course of vaccination. The model is represented as both a set of deterministic ODEs, described in Section 3.1, and as a stochastic network model that explicitly includes heterogeneity in contact transmission, described in Section 3.2. The component states are defined in Table 3-1 and compartment state transitions are illustrated in Figure 3-1. States associated with vaccination using a single vaccine type are explicitly included in the deterministic model. The stochastic model does not currently include these states. In that formulation, vaccination is modeled as a transition into the Recovered state with a probability given by the vaccine efficacy.

Non-Infectious States [1]

S_u	Susceptible and Un-quarantined
S_v	Susceptible, Vaccinated, and Un-quarantined
S_q	Susceptible and Quarantined
E_u	Exposed and Un-quarantined
E_v	Exposed, Vaccinated, and Un-quarantined
E_q	Exposed and Quarantined
R	Recovered

Infectious States

A_u	Infectious, Asymptomatic, and Un-quarantined
A_v	Infectious, Vaccinated, Asymptomatic, and Quarantined
A_q	Infectious, Asymptomatic, and Quarantined
I_u	Infectious, Symptomatic, and Un-quarantined
I_q	Infectious, Symptomatic, and Quarantined
H	Infectious and Hospitalized

Tracking States

D	Cumulative Disease-related Deaths
C	Pending Contacts to Trace

Table 3-1. Compartment Model Population State Definitions

[1]Note: Non-infectious is used to describe states in which viral transmission is not typically assumed to occur. However we can

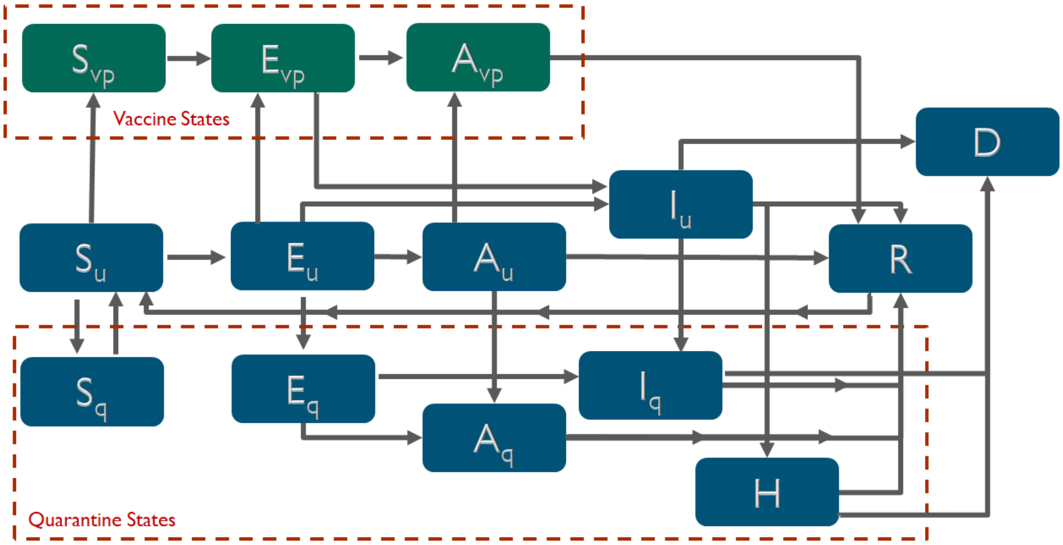

Figure 3-1. Compartment Model States and Transitions

3.1. Deterministic Model

The fundamental dynamics for the deterministic model are defined using a system of ODEs based on basic epidemiological modeling constructs to simulate the spread of infection as population transition rates among a set of exclusive states. States represent a conjunction of disease status, vaccination status, and treatment or control conditions. The model formulation is based on Ngonghala et al. [14] and has been adapted in the following ways:

- Included the possibility of loss of immunity
- Included transition terms that explicitly reflect operational constraints on contact tracing and case identification through community sampling (e.g. resource limits, testing errors).
- Included the potential spread of infections through the exposed population to reflect the two days before symptom onset that a COVID-19 infected individual is infectious.
- Included vaccination of some part of the un-quarantined population with a single course of a vaccine with a given efficacy.

The governing equations, state variables, and parameters are summarized below. For ease in referencing, we provide a description of the model parameters in Table 3-2 and 3-3.

allow for E_u and E_q to generate transmissions because exposed individual is considered contagious in period before symptom onset.

$$\frac{dS_u}{dt} = -\beta\lambda(t)S_u - q_{tS}(t) + \tau_{Sq}S_q + \tau_R R + f_v(t)\nu(t)\frac{S_u}{S_u+E_u+A_u} \tag{3.1a}$$

$$\frac{dS_v}{dt} = f_v(t)\nu(t)\frac{S_u}{S_u+E_u+A_u} - \beta(1-\xi)\lambda(t)S_v \tag{3.1b}$$

$$\frac{dS_q}{dt} = q_{tS}(t) - [1-\theta_{Sq}]\beta\lambda(t)S_q - \tau_{Sq}S_q \tag{3.1c}$$

$$\frac{dE_u}{dt} = \beta\lambda(t)S_u - q_{rE}(t) - q_{tE}(t) - \tau_{Eu}E_u + f_v(t)\nu(t)\frac{E_u}{S_u+E_u+A_u} \tag{3.1d}$$

$$\frac{dE_v}{dt} = f_v(t)\nu(t)\frac{E_u}{S_u+E_u+A_u} + \beta(1-\xi)\lambda_v(t)S_v - \tau_{Ev}E_v \tag{3.1e}$$

$$\frac{dE_q}{dt} = q_{tE}(t) + q_{rE}(t) + [1-\theta_{Sq}]\beta\lambda(t)S_q - \tau_{Eq}E_q \tag{3.1f}$$

$$\frac{dA_u}{dt} = f_a\tau_{Eu} - q_{rA}(t) - q_{tA}(t) - \tau_{Au}A_u + f_v(t)\nu(t)\frac{A_u}{S_u+E_u+A_u} \tag{3.1g}$$

$$\frac{dA_v}{dt} = f_v(t)\nu(t)\frac{A_u}{S_u+E_u+A_u} + f_a\tau_{Ev}E_v - \tau_{Av}A_v \tag{3.1h}$$

$$\frac{dA_q}{dt} = f_a\tau_{Eq}E_q + q_{rA}(t) + q_{tA}(t) - \tau_{Aq}A_q \tag{3.1i}$$

$$\frac{dI_u}{dt} = [1-f_a](\tau_{Eu}E_u + \tau_{Ev}E_v) - q_{rI}(t) - q_{tI}(t) - [\tau_{IuR} + \tau_{IuH} + \tau_{IuD}]I_u \tag{3.1j}$$

$$\frac{dI_q}{dt} = [1-f_a]\tau_{Eq}E_q + q_{rI}(t) + q_{tI}(t) - [\tau_{IqR} + \tau_{IqH} + \tau_{IqD}]I_q \tag{3.1k}$$

$$\frac{dH}{dt} = \tau_{IuH}I_u + \tau_{IqH}I_q - [\tau_{HR} + \tau_{HD}]H \tag{3.1l}$$

$$\frac{dR}{dt} = \tau_{Au}A_u + \tau_{Aq}A_q + \tau_{IuR}I_u + \tau_{IqR}I_q + \tau_{HR}H - \tau_R R \tag{3.1m}$$

$$\frac{dC}{dt} = -q_t(t) + \left[d\phi_\kappa \kappa T_I\left[1-\frac{C}{N}\right]\right][\tau_{Eq}E_q + q_{rA}(t) + q_{rI}(t) + q_{tA}(t) + q_{tI}(t) + \tau_{IuH}I_u] \tag{3.1n}$$

With the force of infection function,

$$\lambda(t) = \frac{\kappa(1-\varepsilon p)(\eta_E(E_u+E_v) + \eta_A(A_u+A_v) + I_u + (1-\theta_{Sq})(\eta_E E_q + \eta_A A_q + I_u + \eta_H H))}{N} \tag{3.1o}$$

the total population, $N = S_u + S_q + S_v + E_u + E_q + E_v + A_u + A_q + A_v + I_u + I_q + H + R$. Noting that we do not assume reduced infectivity of our accidentally vaccinated populations that are already exposed and infected, or eventually become infected due to imperfect efficacy of the vaccine. We let $X = \{S_u, S_q, S_v, E_u, E_q, E_v, A_u, A_q, A_v, I_u, I_q, H, R, C\}$ denote the vector of disease-related compartments along with the quarantine tracing queue state C, since these are the states that govern the propagation of the dynamics. The cumulative disease related death compartment D is used to track the number of total disease related deaths.

$$\frac{dD}{dt} = \tau_{IuD}I_u + \tau_{IqD}I_q + \tau_{HD}H \tag{3.1p}$$

We distinguish this state from the others, since D does not contribute to the governing dynamics.

[2]Note: infected individuals are considered infectious 2 days prior to symptom onset. This can be captured by a marginal influence from the exposure stock.

Disease Transmission Parameters

κ	Effective contact rate (i.e. contacts capable of leading to COVID-19 transmission).	Count/Day
β	Probability of infection per contact.	Probability
T_E	Incubation time for exposed individuals.	Time(Day)
τ_{Eu}	Rate of transition out of the exposed and un-quarantined compartment.	Frequency(Day^{-1})
τ_{Eq}	Rate of transition out of the exposed and quarantined compartment.	Frequency(Day^{-1})
η_E	Relative infectivity of exposed individuals.[2]	Proportion
f_a	Fraction of infectious population that is asymptomatic.	Proportion
τ_{Au}	Rate of transition out of the asymptomatic and un-quarantined compartment.	Frequency(Day^{-1})
τ_{Aq}	Rate of transition out of the asymptomatic and quarantined compartment.	Frequency(Day^{-1})
η_A	Relative infectivity of asymptomatic individuals.	Proportion
τ_{IuR}	Rate of transition from the symptomatic and un-quarantined compartment to recovery.	Frequency(Day^{-1})
τ_{IqR}	Rate of transition from the symptomatic and quarantined compartment to recovery.	Frequency(Day^{-1})
τ_{IuH}	Rate of transition from the symptomatic and un-quarantined compartment to hospitalization.	Frequency(Day^{-1})
τ_{IqH}	Rate of transition from the symptomatic and quarantined compartment to hospitalization.	Frequency(Day^{-1})
τ_{IuD}	Rate of transition from the symptomatic and un-quarantined compartment to deceased.	Frequency(Day^{-1})
τ_{IqD}	Rate of transition from the symptomatic and quarantined compartment to deceased.	Frequency(Day^{-1})
τ_{HR}	Rate of transition from the hospitalized compartment to recovered.	Frequency(Day^{-1})
τ_{HD}	Rate of transition from the hospitalized compartment to deceased.	Frequency(Day^{-1})
η_H	Relative infectivity of a hospitalized infectious individual.	Proportion
τ_R	Rate at which immunity wanes.	Frequency(Day^{-1})
T_I	Average time an infected individual (asymptomatic and symptomatic) is infectious before they go into quarantine.	Time(Day)

Table 3-2. Compartmental Model Disease Related Parameter Definitions

Mitigation Parameters

p	Probability that a contact is protected by PPE.	Probability
ε	Effectiveness of general PPE use in blocking transmission.	Probability
Θ_{Sq}	Effectiveness of quarantine in preventing new infections.	Probability
q_r	Rate of random surveillance sampling.	Frequency(Day^{-1})
N_{trace}	Maximum number of concurrent contact tracings.	Count/Day
q_w	Maximum work rate of a contact tracer.	Work/Day
w_c	Average work required to engage a new contact.	Work/Case
w_q	Average work required to check in with a quarantining contact.	Work/Case
w_v	Average work required to administer a vaccine.	Work/Case
α_q	Responsiveness of contact tracers to workload.	Dimensionless
ϕ_k	Fraction of actual contacts recalled and reported.	Probability
ϕ_c	Fraction of recalled contacts effectively quarantined.	Probability
d	Dispersion of the contact network (i.e. number of distinct individuals per contact event)	Probability
ε_r	Efficiency of random surveillance sampling in selecting exposed/infected	Probability
f_{qe}	Fraction of infected contacts that will be found in the exposed state.	Proportion
e_n	False negative rate of testing.	Probability
e_{ne}	False negative rate fro testing of exposed individuals.	Probability
ξ	Efficacy of vaccine.	Probability
α_v	Scaling factor for bounded exponential application to vaccine distribution.	Dimensionless

Table 3-3. Compartmental Model Mitigation Related Parameter Definitions

Because they are less-common elements of epidemiological models, a more in-depth description is provided for the quarantine functions and the contact tracing queue state C to clarify the mechanistic formulation for the random testing and contact tracing interventions. Since the formulation for the rate at which we simulate contact tracers connecting with cases is dependent on the work related to efforts for vaccinating, we begin this in-depth description with the vaccine distribution mechanistic form.

3.1.1. Vaccine Distribution

Once a vaccine becomes available, distributing the initially limited supplies will raise additional questions about control strategy design. Modeling the distribution of vaccines within our ODE compartment simulations, one consideration will be to assume a fixed nominal rate for which the vaccine would be distributed. By assuming some notional rate of 2% of the susceptible population will be vaccinated, overtime the available population to vaccinate will continuously decrease. The effects of the population decreasing results in a decrease in the number of vaccines distributed over time in the simulated model. This does not reflect the expected nature of initially limited vaccine supplies that will incrementally increase overtime. In place of the nominal fixed rate of vaccine distribution we instead propose to define a function, $v(t)$, that is monotonically increasing and reflective of the practical implications for the

incremental increase of vaccine availability.

With this consideration we run the opposite resource constraint issue. In this case, it is not the limited availability of resources that we would need to constrain. Instead, we will eventually have too many resources to distribute to the depleting available population to distribute these resources.

Using a monotonically increasing vaccine distribution function, $\nu(t)$, (e.g. $\nu(t) = mt + b$ for $m, b > 0$) we can again consider the bounded exponential function to reflect the fraction of the vaccines distributed at time, t, that reflects the current total population available to vaccinate. Assume that the susceptible and un-quarantined population is not the only population to recieve the vaccine. Alternatively, the consideration for unnecessarily vaccinating the un-quarantined exposed and asymptomatic populations is possible if we do not test for infection before administering a vaccine. This implies that the total available population to vaccinate is defined by $N_v(t) = S_u(t) + E_u(t) + A + u(t)$. In this case, when $N_v(t) < \nu(t)$, we would want to reduce the distribution of vaccines to reflect the fraction of the available population to vaccinate with respect to the total amount of vaccines available, $N_v(t))/\nu(t)$. This implies that the bounded exponential with an upper bound of 1 can be used to define this fraction of the vaccine distribution.

$$f_\nu(t) = 1 - \exp\left(\left(-\alpha_\nu\left(\frac{S_u(t) + E_u(t) + A_u(t)}{\nu(t)}\right)\right)\right) \tag{3.2}$$

For an appropriate selection of $\alpha_\nu > 0$, we will simulate the following behavior.

- When the population is greater than the amount of vaccines available, we will simulate distribution of all of the available vaccines.
- When the amount of vaccines exceeds the total population to distribute to, we can take the product, $f_v(t)\nu(t)$, to simulation distribution of only a fraction of the available vaccines.

3.1.2. Contact Tracing

The contact tracing queue state C is the number of contacts provided by newly-identified cases that have yet to be contacted. The rate at which contacts are added to the queue is:

$$C_{\text{new}} = \left[d\phi_\kappa \kappa T_I \left[1 - \frac{C}{N}\right]\right] \left[\tau_{Eq}E_q + q_{rA}(t) + q_{rI}(t) + q_{tA}(t) + q_{tI}(t) + \tau_{IuH}I_u\right]. \tag{3.3}$$

This expression for C_{new} gives us the flow of expected new contacts to trace. The expression,

$$\left[\tau_{Eq}E_q + q_{rA}(t) + q_{rI}(t) + q_{tA}(t) + q_{tI}(t) + \tau_{IuH}I_u\right],$$

includes a term for each of the six unique paths by which an infected person is identified as such (see Figure 3-1), making their contacts new contacts to trace. The sum is the overall rate of recognition of new cases. To estimate the average number of contacts each of these newly-identified infected individuals generate, we included the following factors.

- Potentially infectious contacts will be made at the rate of κ per day.
- There will be a period of time, T_I, for which an infected individual is infectious and unaware, before they become quarantined.

- The average number of contacts is therefore κT_I, of which they will be able to recall and report a fraction ϕ_k
- Some of these contacts may be with the same individual. The dispersion parameter d is the ratio of the number of distinct individuals contacted per total number of contact events.
- C contacts are already known from previously-detected infections. Therefore $[1 - C/N]$ is the probability that a newly-identified contact is not already known and should be added to C.

This results in Equation 3.3 as the rate of arrival of new contacts to trace, based on the number of newly identified infected individuals.

We distill the effects of constraints on the resource required for contact tracing by assuming that contact tracers can each do work at a rate q_w. This potential work rate is applied to the distinct tasks of making initial contacts with the individuals in the queue C, making follow-up contacts to those who have agreed to be quarantined, and supporting vaccine administration. Each task requires a certain amount of work on average, so that the total work demand is:

$$W_{demand}(t) = w_c C(t) + w_q(A_q(t) + I_q(t)) + w_v(S_u(t) + E_u(t) + A_u(t)) \tag{3.4}$$

As the total work demand approaches the capacity of available workers, the latter will become limiting. This effect is modeled using an exponential relationship, $g : \mathbb{R} \to \mathbb{R}$, between work demanded and work applied to all tasks:

$$g(W_{demand}(t)) = q_w N_{trace}(1 - exp(-\alpha_q W_{demand}(t))) \tag{3.5}$$

Work is applied to tasks in proportion to demand. Specifically the work applied to tracing new contacts is:

$$\frac{w_c C(t)}{W_{demand}(t)} (g(W_{demand}(t))) \tag{3.6}$$

so that the rate of engagement with new contacts (and the rate of depletion of the queue C) is:

$$q_t(t) = \frac{C(t)}{W_{demand}(t)} (g(W_{demand}(t))) \tag{3.7}$$

Of these, a fraction β will have resulted in transmission (and $1 - \beta$ will not). The rate of quarantined susceptible is therefore approximately

$$q_{tS} = (1 - \beta)\phi_c q_t \tag{3.8}$$

where ϕ_c is the fraction of individuals successfully contacted and quarantined during tracing.

As we consider the infected contacts, we ask the question: where will these individuals be with respect to disease progression? If tracing is rapid and the initiating infected case arrived early in their progression, many secondary cases might still be in the exposed state. Conversely, if the precipitating case has been infected for many days then their induced cases may already be infectious themselves or possibly even recovered. An optimistic limiting assumption would be that all are still in the exposed state. Alternatively, we can use the residence time in the exposed and infected states to apportion quarantines among states:

$$f_{eq} = \frac{T_E}{T_E + T_I}$$

The potential rates of quarantine from the exposed, unquarantined symptomatic, and asymptomatic populations are approximately,

$$q_{tE} = \beta\phi_c q_t(1-e_n)f_{eq} \tag{3.9a}$$
$$q_{tA} = \beta\phi_c q_t(1-e_n)f_a(1-f_{eq}) \tag{3.9b}$$
$$q_{tI} = \beta\phi_c q_t(1-e_n)(1-f_a)(1-f_{eq}). \tag{3.9c}$$

These equations treat the false negative rate of testing as a leakage or inefficiency applied to the *inflows* to the quarantine states. In the case of exposed individuals, we assume that by the time a test is administered to determine whether the person should remain quarantined, that enough time will have passed for the likelihood of receiving a false negative result to be approximately equal to that of a symptomatic and infected individual.

3.1.3. Random Testing

Surveillance testing is conducted at a rate of q_r. If the surveillance is random (uninformed), all individuals in the un-quarantined populations will be equally likely to be selected. We use an efficiency parameter ε_r, in the spirit of Siddarth and Wyle [18], to examine the value of some unspecified mechanism that might improve targeting, with $\varepsilon_r = 0$ corresponding to random selection and $\varepsilon_r = 1$ corresponding to perfect targeting of only exposed and infectious people. Rates of identification and quarantine due to random testing are then:

$$q_{rE} = q_r(1-e_{ne})\frac{E_u}{P_{\text{eff}}(t)} \tag{3.10a}$$
$$q_{rA} = q_r(1-e_n)\frac{A_u}{P_{\text{eff}}(t)} \tag{3.10b}$$
$$q_{rI} = q_r(1-e_n)\frac{I_u}{P_{\text{eff}}(t)} \tag{3.10c}$$

where P_{eff} is the effective sample population, given by the efficiency

$$P_{\text{eff}}(t) = (1-\varepsilon_r)(S_u+R)+E_u+A_u+I_u.$$

This time we consider the false negative rate for exposed asymptomatic may be larger than for the infectious. In contrast to the test administered to release an exposed individual from quarantine, random tests can be administered at any time during the incubation period and so can miss identification of an infected individual with greater likelihood than if that individual was in an infectious state.

The behavior of the deterministic model has been explored both through analysis of the defining ODEs and through simulation. Section 4 summarizes some findings from that work.

3.2. Stochastic Network Model

A stochastic network-based representation of the deterministic model was developed to include stochastic effects and heterogeneity. This work builds on the stochastic network model developed by McGee [11]. The network represents possible contacts between people and can therefore be used couple the models of

transmission and contact tracing processes, as well as to associate contacts with particular locations and activities. These features can only be roughly approximated in compartment models, for example in estimating the proportion of contacts that will be found in particular disease states in 3.9. This section describes how network structure influences key process rates in the model.

Contact networks can be generated in various ways, for example by sampling an assumed stochastic form or by construction from mobility data. Methods to a estimating network structure from near-real-time cell phone tracking data are described in Section 6. Explicit representation of the contact network offers several advantages over the assumption of uniform mixing embedded in compartment models. First, the network integrates variability in the size and complexity of individuals' contact structures that can be used to define contact tracing. By defining location-mediated contact networks, the model can be used to examine sampling strategies focused on specific kinds of locations or activities. Second, the same network allows for the evaluation of protective measures designed to decrease transmission in specific location types. For example, specific social distancing measures and business constraints can be tested by modifying the network or modifying edge weights on the network.

It is important to note that the network is not intended to represent individuals and track their exact interaction through time, as might be done with an agent-based model. The network is intended to represent interactions that are representative of a population at a scale that is relevant to decision makers. This includes, for example, the interactions people have at schools, businesses, and places of work. These interactions can be defined based on demographics, types of businesses, and other spatial considerations (e.g., rural vs urban settings) within the model domain.

Unlike the Deterministic Model, the Stochastic Network Model tracks the state of each node, i, in the network through time, $X_i(t)$. With the exception of the vaccination states, it uses the same state definitions and transition times as the deterministic model, along with an explicit structure of a contact network. The contact network can include edge weights that describe the level of interaction between two nodes. The number of social interactions is defined by the node degree, which can be weighted using edge weights. For example, people in strict quarantine have a small node degree while super-spreaders have a large node degree.

The explicit contact network underlying the Stochastic Network Model enables a finer-grained representation of the contact tracing process than the Deterministic Model permits. The Stochastic Network Model includes a dynamic contact list that indicates nodes that should be considered for contact tracing. When a node transitions into a controlled state (A_q, I_q, or H) the neighboring nodes, defined by the network structure, are added to a contact list. The fraction of contacts that can be recalled, ϕ_k, and the proportion of contacts that will comply, ϕ_c, are used to down-select the contacts that are actually added to the list. The probability of a connected node being nominated is proportional to the weight connecting that node to the newly-transitioned node. Furthermore, a threshold can be used to determine "close contacts" based on the values in edge weights.

The Deterministic Model's state variable C can only record the number of contacts to be traced, which are allocated among the various populations based on assumptions about contact structure (Equations (3.8) & (3.9)). The network structure, by contrast, allows specific individuals involved with potential transmission pathways to be identified. The contact list can also store attributes about each contact, including the time the contact was added to the list, the edge weight associated with the transition (defining the interaction strength between those two nodes), and the node degree of the neighboring node. These attributes can be used to evaluate prioritization or targeting strategies in contact tracing. The time at which the nodes are

added to the contact list can also be used to remove contacts if they are not engaged within a certain amount of time. Nodes that transition into a controlled state, for any reason, are also removed from the contact list.

The rates of quarantine due to contact tracing are based on the same equations used in the deterministic model but also include the contact list $C_i(t)$, as shown below. In general, $C_i(t) = 1$ if node i is in the contact list and 0 otherwise. As noted above, additional node attributes can also be used to define the contact list, for example the edge weight or the node degree. If these attributes are used to include contact tracing prioritization, $C_i(t)$ should be scaled between 0 and 1. N_c is the number of people on the contact list, and $\delta_{X_i(t)=Z} = 1$ if the state of X_i(t) is Z and 0 otherwise. The Stochastic Network Model uses the same work demand, g, and exponential relationship between work demand and work applied to contact tracing, f, described in Equation 3.4 and 3.5 to compute q_t.

$$N_c(t) = |C_i(t)| \quad \forall C_i(t) > 0 \tag{3.11a}$$

$$q_t(t) = f\frac{N_c(t)}{g} \tag{3.11b}$$

$$q_{tS,i}(t) = \phi_c q_t(t) C_i(t) \delta_{X_i(t)=S_u} \tag{3.11c}$$

$$q_{tE,i}(t) = \phi_c q_t(t)(1-e_n) C_i(t) \delta_{X_i(t)=E_u} \tag{3.11d}$$

$$q_{tI,i}(t) = \phi_c q_t(t)(1-e_n) C_i(t) \delta_{X_i(t)=I_u} \tag{3.11e}$$

$$q_{tA,i}(t) = \phi_c q_t(t)(1-e_n) C_i(t) \delta_{X_i(t)=A_u} \tag{3.11f}$$

The rates of quarantine due to random testing are also based on the corresponding equations used in the Deterministic Model, as shown below.

$$q_{rE,i}(t) = q_r(1-e_{ne})\frac{\delta_{X_i(t)=E_u}}{P_{eff}(t)} \tag{3.12a}$$

$$q_{rI,i}(t) = q_r(1-e_n)\frac{\delta_{X_i(t)=I_u}}{P_{eff}(t)} \tag{3.12b}$$

$$q_{rA,i}(t) = q_r(1-e_n)\frac{\delta_{X_i(t)=A_u}}{P_{eff}(t)} \tag{3.12c}$$

In addition to transmission along network connections, infection can also be transmitted between individuals chosen at random regardless of network structure. The force of infection, $\lambda_i(t)$, therefore has a global and a local network component. The global component is defined by the same equation used in the Deterministic Model. For the global component, the effective contact rate κ can be defined using the average node degree of the contact network. The local network component, $\lambda_{N,i}$ uses the contact network to define the probability of transmission based on the state of neighboring nodes. The parameter Θ_p defines the interaction between global and local transmission. When $\Theta_p = 0$, transmission is governed only by local direct contacts, while $\Theta_p = 1$ disregards network structure when modeling disease transmission and so represents a uniformly mixed population.

$$\lambda_{N,i}(t) = (1-\varepsilon_p)[(\eta_E \delta_{X_i(t)=E_u} + \eta_A \delta_{X_i(t)=A_u} + \delta_{X_i(t)=I_u}) + (1-\Theta_{Sq})(\eta_E \delta_{X_i(t)=E_q} + \eta_A \delta_{X_i(t)=A_q} + \delta_{X_i(t)=I_q} + \eta_H \delta_{X_i(t)=H})] \tag{3.13a}$$

$$\lambda_i(t) = \Theta_p \lambda(t) + (1-\Theta_p)\lambda_{N,i}(t) \tag{3.13b}$$

The Stochastic Network Model defines a transition probability between states, for example, the probability that node i transitions from state S_u to E_u. The underlying transition probabilities are consistent with the

rate terms used in the Deterministic Model. The contact network is used to define $q_{tS,i}(t)$, $q_{tE,i}(t)$, $q_{tI,i}(t)$, $q_{tA,i}(t)$, and the local network component of $\lambda_i(t)$. While β is generally a constant, it can also be defined per node or per edge. When using a weighted contact network that includes edge weights to define the interaction between two nodes, the edge weight can be used to modify beta. This provides a mechanism for modeling the effect of NMIs, for example by reducing transmission probability to represent use of PPE during specific kinds of interactions.

$$
\begin{aligned}
Pr(X_i(t) = S_u \to E_u) &= \beta \lambda_i(t) \delta_{X_i(t)=S_u} && (3.14a)\\
Pr(X_i(t) = S_u \to R) &= f_v * \xi_v \delta_{X_i(t)=S_u} && (3.14b)\\
Pr(X_i(t) = S_q \to E_q) &= (1-\Theta_{Sq})\beta \lambda_i(t) \delta_{X_i(t)=S_q} && (3.14c)\\
Pr(X_i(t) = E_u \to A_u) &= f_a \tau_{Eu} \delta_{X_i(t)=E_u} && (3.14d)\\
Pr(X_i(t) = E_u \to I_u) &= (1-f_a)\tau_{Eu} \delta_{X_i(t)=E_u} && (3.14e)\\
Pr(X_i(t) = E_q \to A_q) &= f_a \tau_{Eq} \delta_{X_i(t)=E_q} && (3.14f)\\
Pr(X_i(t) = E_q \to I_q) &= (1-f_a)\tau_{Eq} \delta_{X_i(t)=E_q} && (3.14g)\\
Pr(X_i(t) = I_u \to R) &= \tau_{IuR} \delta_{X_i(t)=I_u} && (3.14h)\\
Pr(X_i(t) = I_u \to H) &= \tau_{IuH} \delta_{X_i(t)=I_u} && (3.14i)\\
Pr(X_i(t) = I_u \to D) &= \tau_{IuD} \delta_{X_i(t)=I_u} && (3.14j)\\
Pr(X_i(t) = A_u \to R) &= \tau_{Au} \delta_{X_i(t)=A_u} && (3.14k)\\
Pr(X_i(t) = H \to R) &= \tau_{HR} \delta_{X_i(t)=H} && (3.14l)\\
Pr(X_i(t) = H \to D) &= \tau_{HD} \delta_{X_i(t)=H} && (3.14m)\\
Pr(X_i(t) = S_u \to S_q) &= q_{tS,i}(t) && (3.14n)\\
Pr(X_i(t) = E_u \to E_q) &= q_{rE,i}(t) + q_{tE,i}(t) && (3.14o)\\
Pr(X_i(t) = I_u \to I_q) &= q_{rI,i}(t) + q_{tI,i}(t) && (3.14p)\\
Pr(X_i(t) = A_u \to A_q) &= q_{rA,i}(t) + q_{tA,i}(t) && (3.14q)\\
Pr(X_i(t) = I_q \to R) &= \tau_{IqR} \delta_{X_i(t)=I_q} && (3.14r)\\
Pr(X_i(t) = I_q \to H) &= \tau_{IqH} \delta_{X_i(t)=I_q} && (3.14s)\\
Pr(X_i(t) = I_q \to D) &= \tau_{IqD} \delta_{X_i(t)=I_q} && (3.14t)\\
Pr(X_i(t) = A_q \to R) &= \tau_{Aq} \delta_{X_i(t)=A_q} && (3.14u)\\
Pr(X_i(t) = S_q \to S_u) &= \tau_{Sq} \delta_{X_i(t)=S_q} && (3.14v)\\
Pr(X_i(t) = R \to S_u) &= \tau_R \delta_{X_i(t)=R} && (3.14w)\\
Pr(X_i(t) = any \to S_u) &= \nu \delta_{X_i(t) \neq D} && (3.14x)
\end{aligned}
$$

Equation 3.14b models the effect of vaccination as a direct transition from the susceptible to recovered state. The vaccination rate f_v is a function of the number of vaccines administered per unit time n_v and the total population targeted for vaccination, which might include exposed and asymptomatic individuals as well as susceptible individuals.

$$f_v = \frac{n_v}{S_u + E_u + I_a} \tag{3.15}$$

The model also includes the ability to change the contact network (and other parameters) at specific points

in time. This capability allows the model to include distinct changes in behavior that can occur due to policy decisions regarding social distancing or business closures.

Figure 3-2 illustrates a comparison between the Deterministic and Stochastic Network Model under equivalent conditions, i.e. with a random network designed to produce bulk interaction rates equivalent to those in the Deterministic model. The example uses $\kappa = 6$ and a contact network with the same value for average node degree. In the Stochastic Network Model using Θ_p=0.5, such that 50% of transmission is governed by the network and 50% is governed by global uniform mixing. This results verifies that the Stochastic Network Model can be configured to act as a discrete version of the Deterministic model. However its real value is in exploring the effects of network structures that the Deterministic model cannot represent, whether those are abstract networks based on theoretical considerations (**??**) or on behavioral data (6).

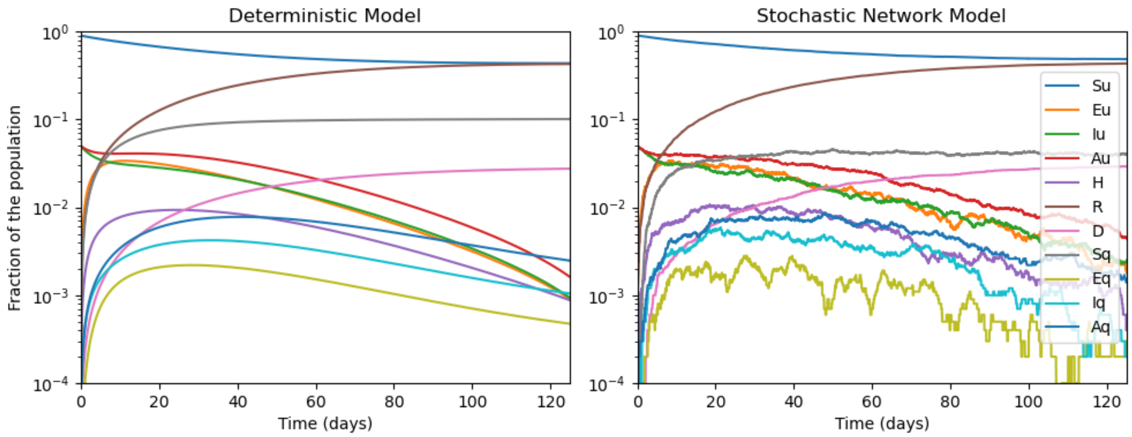

Figure 3-2. Deterministic and Stochastic Network Model Comparison: under equivalent conditions.

4. DYNAMICAL SYSTEM ANALYSIS

The mathematical formulations for the governing system of differential equations defined by Equations (3.1) is the basis for the stochastic model's individual transitions between identified disease related state compartments and their corresponding quarantine states. To ground our understanding of the layered network analysis that allows us to provide higher fidelity and more localized contact rates, we have analyzed dynamical properties related to the mechanistic formulation we have structured for the governing dynamics. We look to understand the number of physically-relevant equilibria that exist, their stability, and any corresponding bifurcations. This type of analysis has been explored for decades and a more in-depth description can be found in Hethcote's article, *The Mathematics of Infectious Disease* [6], or Martcheva's book, *An Introduction to Mathematical Epidemiology* [9]. For the purposes of our discussion, we are simply providing a summary of the analysis and different model formulations.

From the perspective of SEIR (Susceptible-Exposed-Infected-Recovery) dynamics (i.e. modeling the epidemiology independent of mitigation strategies) there are two fundamental formulations that determine whether the system will have one or two physically-relevant equilibria. *Epidemic* models have one equilibrium. Due to the somewhat closed nature of the dynamics, we will see that a long run analysis will result with the disease eventually eradicated through herd immunity. We refer to this equilibrium as the 'disease-free' equilibrium. In contrast to the epidemic models, which tend to be used more for short term forecasting, *endemic* models include rates into and out of the system that represent birth and non-disease related deaths. Endemic models are assumed to be more relevant for long term analysis (on the order of years). Under the endemic model formulation, we expect two equilibria. We will still have a characterization for a disease-free equilibrium, but with the addition of a new stock of susceptible individuals flowing in there is the possibility of stabilizing at a second endemic equilibrium. Only one of these equilibria will be stable. The determination for which equilibrium is stable, is an indicator for whether to expect an epidemic or eradication. This relates to the assessment of the secondary infection rate, R_0. Applying mathematical theory for dynamical systems analysis, bifurcation analysis can be used to determine the transfer of stability from the disease-free equilibrium to the endemic equilibrium. This results in a symbolic formulation of R_0, in terms of the parameters of the SEIR model, as defined by the governing dynamics.

We want to emphasize that the goal of our analysis is to determine the conditions under which we will be able to implement controls to mitigate the number of new infections. This can tie directly into a bifurcation analysis that would provide an analytical formulation for guiding a system to cross the bifurcation boundary to a more desirable outcome, assuming our system is tending towards an undesirable endemic equilibrium. Because we have now coupled the control mechanisms with the disease transmission states, we may identify an increased number of equilibria. Determining where the additional equilibria surface as a function of the coupled control mechanisms is essential to the analysis of controlling a dynamical system.

A simplified version of Equations (3.1) defined the original epidemiological model we considered in the following analysis. Specifically, the original system of equations had the following simplifications in comparison to the current model:

- We had not yet introduced vaccination compartments at the time of this analysis.
- The rate at which the contact tracing queue state, C, depleted, was defined by the following.

$$q_t(t) = \frac{min(C(t), N_{trace})}{T_{trace}} \tag{4.1}$$

Where T_{trace} defined the nominal time it would take a contact tracer to reach each contact.

With this formulation and under conditions of no mitigations, the model will behave as documented by Hethcote, with convergence to the one disease-free equilibrium. Adding the mitigation strategies, we intuitively expect that early identification and isolation of infected individuals with quarantine of the potentially infected population would simply expedite convergence to the disease-free equilibrium. To determine what parameters most influence time to convergence, we explored sampling methods that allow us to better characterize the anticipated outcomes for various parameter selections (a full inspection of this analysis is provide in Section 5).

Can we say mathematically what the minimum requirements are for the number of contact tracers and randomized testing rate, based on the current stage of a pandemic to mitigate secondary infections? To answer this question, we began by exploring long run simulations of the state dynamics, but with a particular focus on the total cumulative deaths and the contact tracing queuing population. An example result shows that, even under the epidemic model formulations, we can identify a threshold for initial prevalence at which the addition of one more infected individual will tax the contact tracers to the point that they are no longer able to keep up with the contact tracing queue. Table 4-1 indicates the fixed parameter values used to run the simulations on either side of the threshold. Figures 4-1 and 4-2 illustrate the vastly different outcomes in those simulations when initial prevalence goes from 296 to 297. The total population was set to one million, implying that the difference between controllable and uncontrollable prevalence represents a marginal fraction of one percent of the population.

This split in behavior that we have identified is not an example of an equilibrium bifurcation, although it does indicate that there is a boundary in the phase portraits for the cumulative death state. Further investigation is required to analytically characterize a relationship between initial prevalence (along with additional parameters) and the number of contact tracers and random tests needed to maintain control of secondary infections.

Initial Population	$1M$	N_{trace}	1000	q_r	10000
κ	10	β	0.03	f_a	0.6
ϕ_k	1	ϕ_c	1	εp	0
η_E	0	η_A	0.5	η_H	0.1
Θ_q	0.7	ε_r	0	T_{trace}	1
e_n	0.05	e_{ne}	0.05	d	0.3
$1/\tau_{Sq}$	14	$1/\tau_{Eu} = 1/\tau_{Eq}$	14	$1/\tau_{Au} = 1/\tau_{Aq}$	14
$1/\tau_{IuR} = 1/\tau_{IqR}$	11.3	$1/\tau_{IuH} = 1/\tau_{IqH}$	28	$1/\tau_{IuD} = 1/\tau_{IqD}$	256
$1/\tau_{HR}$	11.3	$1/\tau_{HD}$	34	$1/\tau_R$	1600

Table 4-1. Numerical Values for Deterministic Simulation Parameters: these are the fixed parameters used to simulate the 1000 day forecasts provided in Figures 4-1 and 4-2

We note that Figure 4-2 appears to show a steady state of approximately 14000 individuals in quarantine after the disease has been eradicated in the population. This is a consequence of the rapid build up of the

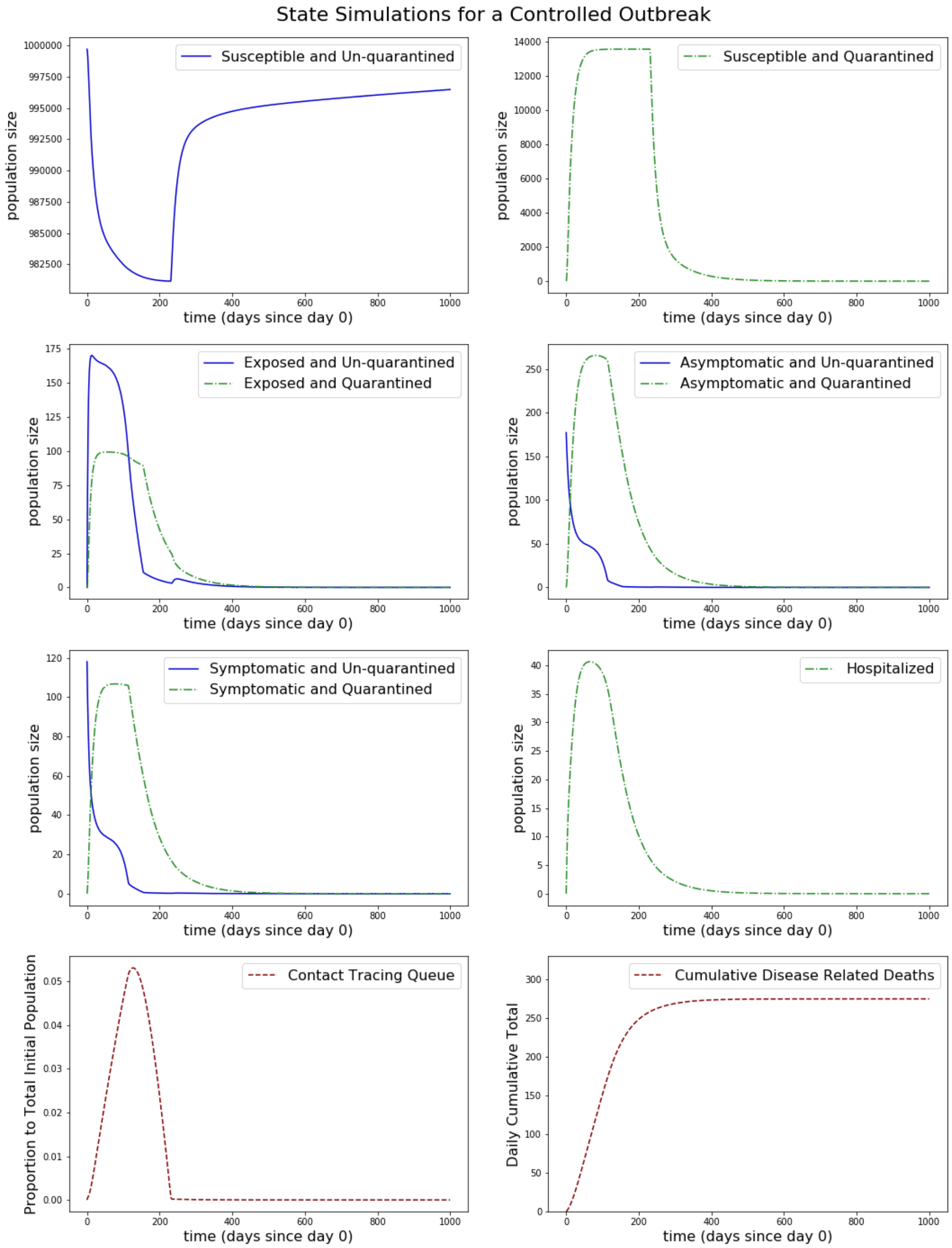

Figure 4-1. State simulations for a controlled outbreak with initial prevalence of 296 out of 1,000,000 : time horizon of 1000 days with parameters defined in Table 4-1.

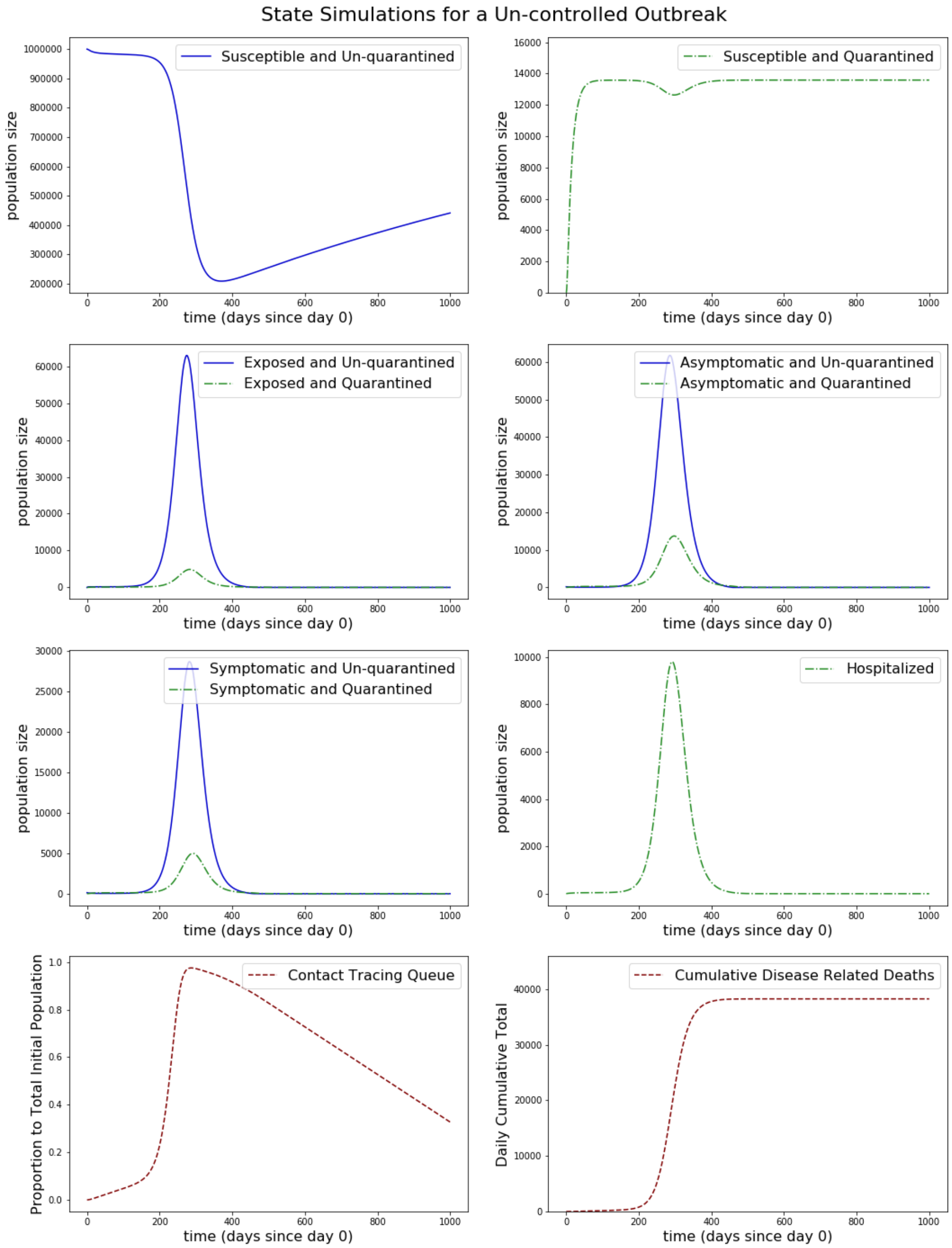

Figure 4-2. State simulations for an un-controlled outbreak with initial prevalence of 297 out of 1,000,000 : time horizon of 1000 days with parameters defined in Table 4-1.

contact queue, absence of expiration of queued contacts, and lack of higher-order controls on the quarantine process. and The model dynamics could be elaborated to remove this artifact, but those changes would not change the underlying dynamics that overwhelm the system's capacity.

5. NETWORK STRUCTURE SENSITIVITY

The Deterministic and Stochastic Network Model formulations are based on a common state transition model (Figure 3-1) which describes the possible conditions of individuals with respect to both disease state and administrative controls on individuals (quarantine and hospitalization). The ODE model approximates the state of a population through continuous variables describing the number of individuals in each state. Disease transmission involves interactions among susceptible and infectious individuals. The ODE model does not differentiate individuals, and interactions rates can only depend on the numbers of individuals in the relevant states.

The network model resolves the state of each node, which represents an individual. Contingencies of disease spread, arising from small numbers of individuals in infectious states for example, can arise in this formulation. This allows designs for disease control to be tested for robustness against stochastic variability. Likely more significant for our purpose, the network explicitly represents relationships among individuals in the form of edges. These relationships can be used to define the workload of contact tracers, as well as the power of using properties of the relationship (such as the location in which it occurs) to guide control actions.

As an aid to designing containment systems, the network representation has advantages over the population-level ODE formulation. It allows heterogeneity in the distribution of contacts across individuals in the population. This heterogeneity can be important for understanding both disease transmission, contact tracing, and surveillance sampling. It also imposes causal relationships among the states of connected individuals that can only be roughly approximated through biasing sampling under the population-level ODE formulation. These relationships are important when modeling the effect of contact tracing.

The Stochastic Network Model was used to understand the influence of network structure on control system requirements through a set of sensitivity studies. Each used a range of values for some key epidemiological parameters, listed in Table 5-1 below. Control parameters corresponding to alternative levels of social distancing (via κ) and different levels of resource commitments to contact tracing (N_{trace}), and random surveillance sampling (q_r) were systematically varied by combining the discrete values shown in Table 5-2. The specified doubling times reflect potential spread in a naive population under nominal contact rates (defined as $\kappa = 10$). (Note: These studies were conducted with a version of the model that did not include vaccination and that did not account for contact tracers' time in maintaining contacts with existing quarantined people. These recent extensions should not change the sensitivities identified below.)

Figure 5-1 shows a set of results generated with the Deterministic Model. These serve as a reference for measuring the effect of explicit representation of contact structure using the Stochastic Network Model, as well as providing general insights about the interaction of control measures and epidemiological parameters to determine consequences. A population of 10,0000 is initialized with a random number of infected individuals (Uniform from 1 to 100) and simulated for 300 days. Among the epidemiological parameters we varied, the doubling time under unmitigated conditions has the largest influence on the key output measures, among which is the number of deaths from the disease. Each of the panel plots therefore shows the calculated number of deaths on the *y*-axis versus unmitigated doubling time on the *x*-axis.

Sampled Epidemiological Parameters

Parameter	Units	Distribution Description
Doubling Time	Days	Triangular from 1.75 to 5, triangular with mode of 2.5
fa	[.]	Uniform from 0.4 to 0.6
T_e	Days	Uniform from 5.5 to 10.5
Initial Infected	[.]	Uniform from 1 to 100

Table 5-1. Epidemiological Parameter Variance for Sensitivity Study

Sampled Epidemiological Parameters

Parameter	Units	Values
N_{trace}/T_{trace}	contacts traced per day	1, 10, 100, 1000
q_r	samples per day	100, 1000, 3000
κ	contacts per day	2, 4, 6, 8, 10

Table 5-2. Control Parameter Variance for Sensitivity Study

Doubling time was varied from 1.75 to 5 days in the simulations; larger values typically have small consequences so plots focus on the interval from 1.75 to 3 . Other output metrics are the number hospitalized, the number of samples analyzed, and the number of outstanding contacts to trace.

Three primary control mechanisms were considered: some degree of social distancing reflected as suppression of the contact rate κ below its nominal value of 10 per day; N_{trace}, the capacity for tracing contacts of newly-discovered cases ranging in powers of 10 from 1 to 1000 per day; and q_r, the rate of testing randomly-selected members of the population at rates of 100, 1000, and 3000 per day. The results for each combination of contact tracing resources and random sampling are shown on a separate panel in Figure 5-1, with alternative values for κ distinguished by color and symbol in each panel.

Low levels of control, such as the conditions plotted in the upper-left panel with κ=10, lead to effectively unmitigated spread and the death of approximately 5% of the population. Various combinations of contact reduction, contact tracing, and surveillance sampling can effectively limit spread and suppress deaths over the 300-day simulation period to a much smaller portion of the population. The analysis is intended to let decision-makers understand the interplay among the control mechanisms so that they can select a mixture of controls that fits within their constraints and risk tolerance. For example, a pure social-distancing policy leading to a nominal contact rate of 40% leads to fewer than 100 deaths in all simulations. To accommodate a smaller suppression of contacts, for example reduction to 80% of nominal, a mix of contact tracing and random surveillance sampling is required. Model results indicate that contract tracing alone (top row) is unable to reduce deaths below 100 with high confidence. (We assume that approximately 50% of contacts are identified and controlled). In contrast, surveillance sampling alone (right column) can reduce deaths to this extent, however that strategy requires 30% of the population to be tested every day. Some combination, which exploits all of the contact information provided by new cases quickly, but also monitors for cases that can't be reached through tracing, is evidently needed. As the analysis in Section 4 shows, system design can rapidly become overwhelmed if it is undersized for the number of outstanding cases.

The Stochastic Network Model provides more insight for designing control strategies because it explicitly represents the contact networks mediating much disease transmission, and whose partially-observed linkages are navigated by contact tracers. The model is more computationally demanding than the Deterministic Model, and so simulations were confined to regions of the control space bracketing

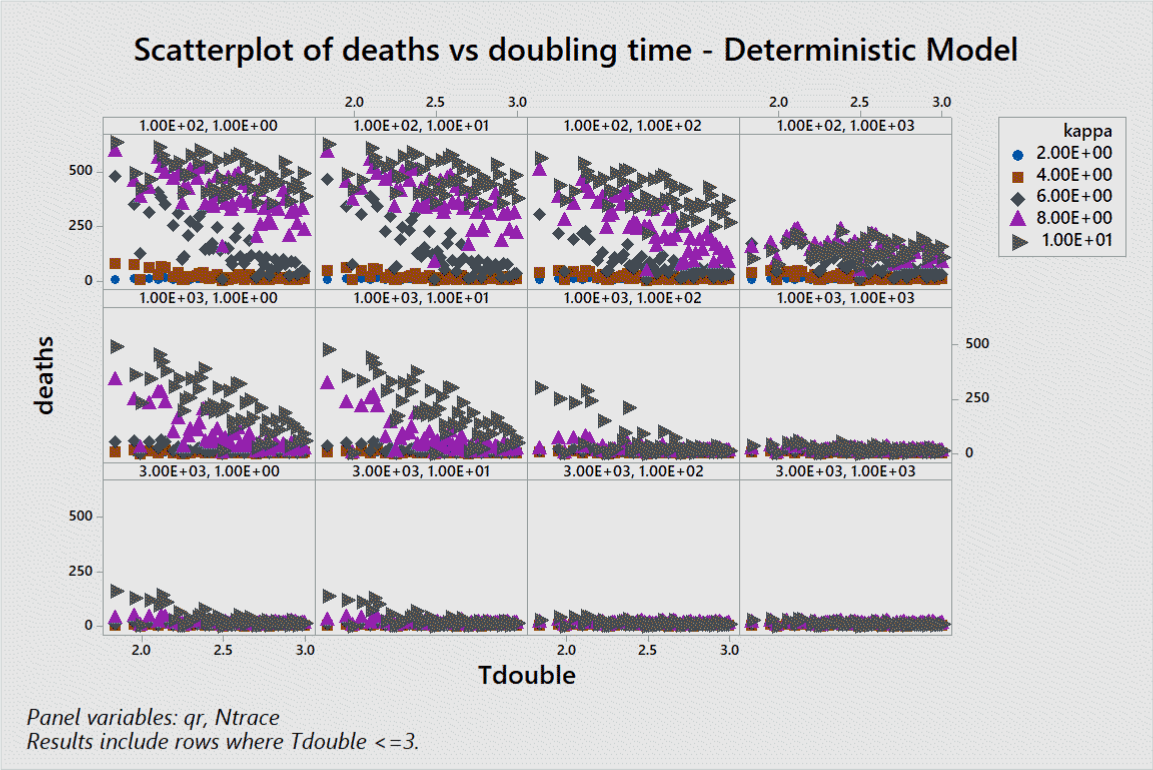

Figure 5-1. Deterministic Model-derived deaths as a function of several control parameters with uncertainty in epidemiological parameters.

transitions in calculated deaths as indicated by results from the Deterministic Model. In all simulations, the same set of 100 sampled parameter values were used to provide clearer cross-mode comparisons.

Three types of idealized networks were used to give insight into the role of structured contacts on shaping disease progression and control. First, disease transmission was entirely governed by random selection of node pairs, independent of network structure. This configuration corresponds to a discretized stochastic version of the Deterministic Model. The two formulations differ only in the use of the network structure to guide contact tracing, which under the assumptions of this configuration will be uninformative. The second network structure assigns a uniform degree to each node, and randomly connects pairs given that constraint. The third network type uses an algorithm that generates a Barabasi-Albert (BA) style network via preferential connection, then adjusts node degrees using an exponential distribution filter [11]. For each network type, a series of networks was generated having an average degree equal to κ. The interaction frequencies are the same for each connection, so that contact heterogeneity is determined just by the network structure. Simulations using the uniform and BA network structures disabled global transmission, so that transmissions only occurred through network edges.

To facilitate comparison of results from the Deterministic and Stochastic Network Models, Figure 5-2 shows results from the Deterministic Model over the central values for contract tracing, the lowest values for surveillance sampling, and the largest values for contact rate. Figure 5-3 shows the corresponding Network Model results in which all contacts occur through random transmissions. The models produce similar results, and would suggest the same conclusions regarding the kinds of controls needed to limit deaths. Deaths in the Deterministic Model appear systematically larger than in the Network Model, especially for parameter values leading to slow transmission (larger doubling time, smaller κ). This may be

due to the possibility of disease extinction in the discrete Network Model, however this has not been confirmed.

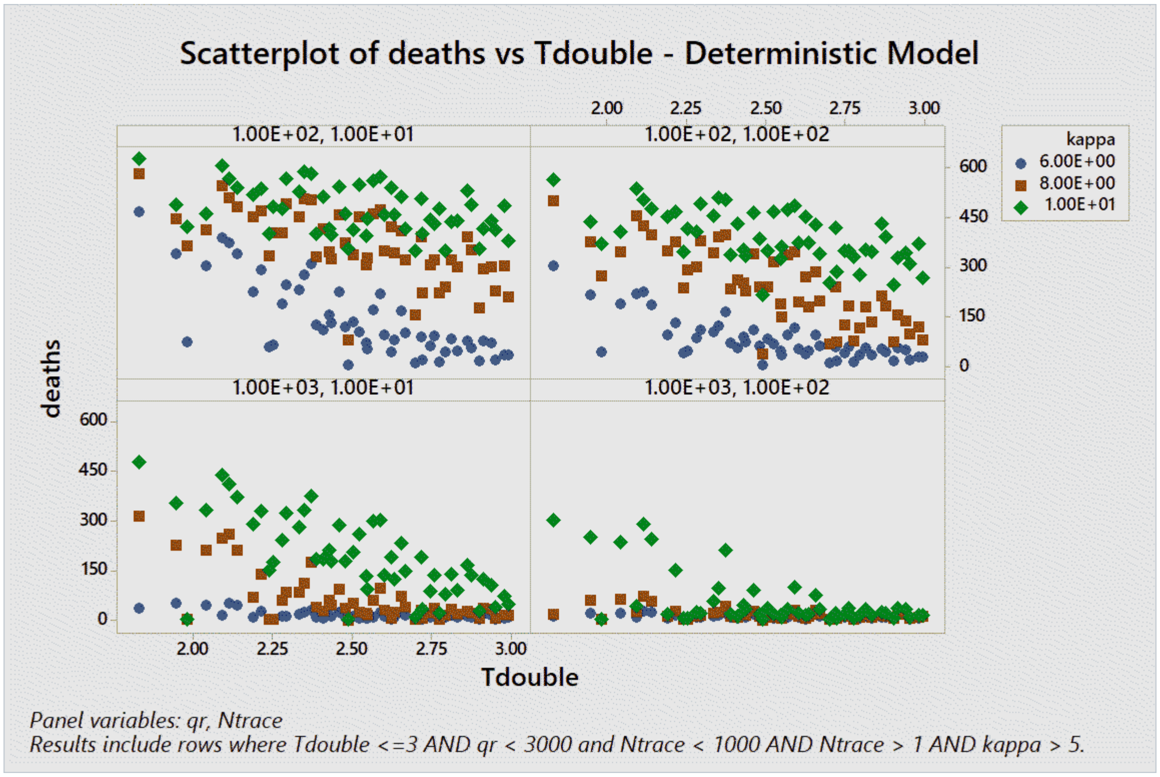

Figure 5-2. Deterministic Model-derived deaths as a function of several control parameters with uncertainty in epidemiological parameters (subset of results shown in Figure 5-1).

Figure 5-4 shows results using networks in which each node has the same number of connections (equal to κ). This structure leads to a substantially lower number of deaths for all parameter values and control conditions. The tendency for heterogeneity in network connection degree to foster disease spread (using a uniform degree) has been reported previously (e.g. [10]).

Results from networks generated through the modified BA algorithm are shown in Figure 5-5. In comparison to the case of global transmission, there are generally fewer deaths when tracing and surveillance efforts are low (upper left panel), however both tracing and surveillance appear to be less effective in controlling outbreak based on the downward shifts achieved by increasing tracing (right column) and surveillance (lower row). One motivation for developing the Stochastic Network Model is to allow evaluation of the effectiveness of different strategies for deploying testing and tracing resources. The concept of targeting surveillance on individuals more likely to have many contacts can be explored by making probability of selection for testing proportional to node degree. Figure 5-6 shows the result of that modification. Increasing surveillance sampling (from the top to bottom row) is clearly more effective when that sampling can be targeted. To better test the potential for this kind of targeting, and to make it potentially operational, prioritization might be based on a person’s employment or on their connection to specific locations or events. The mobility-derived networks developed as part of our research enable this important next step. Section 6 discusses that work.

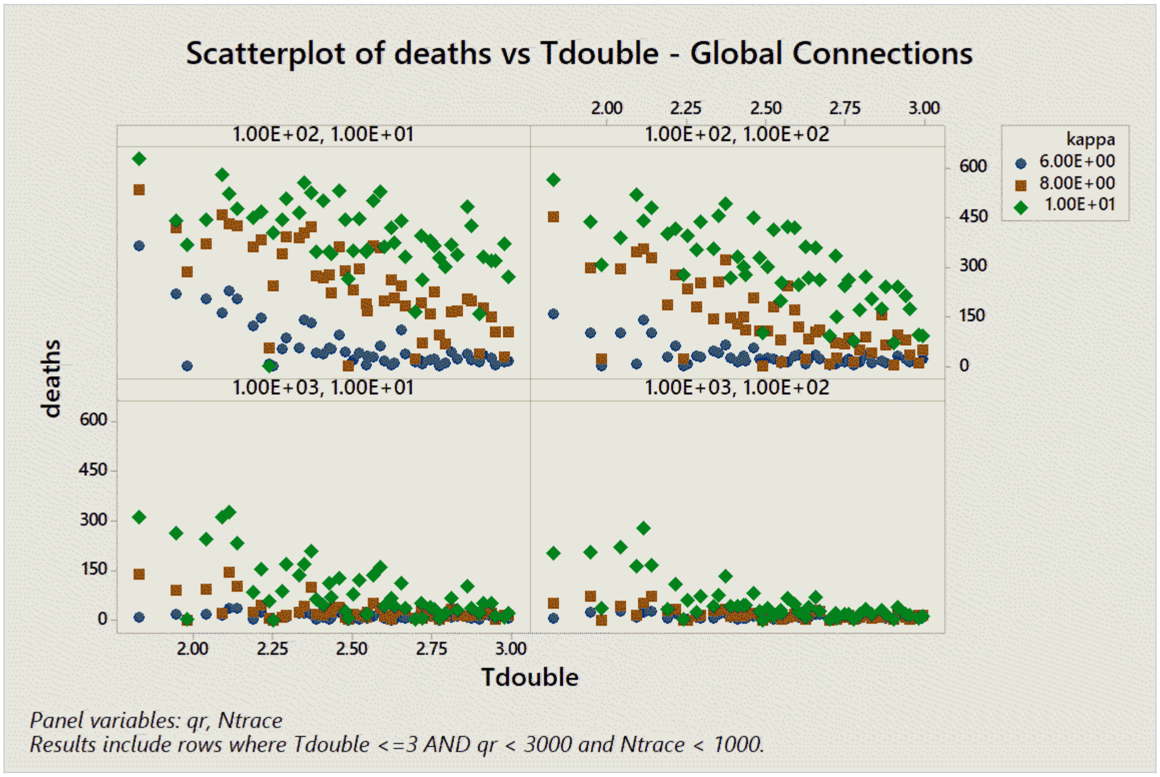

Figure 5-3. Stochastic Network Model-derived deaths using global disease transmission.

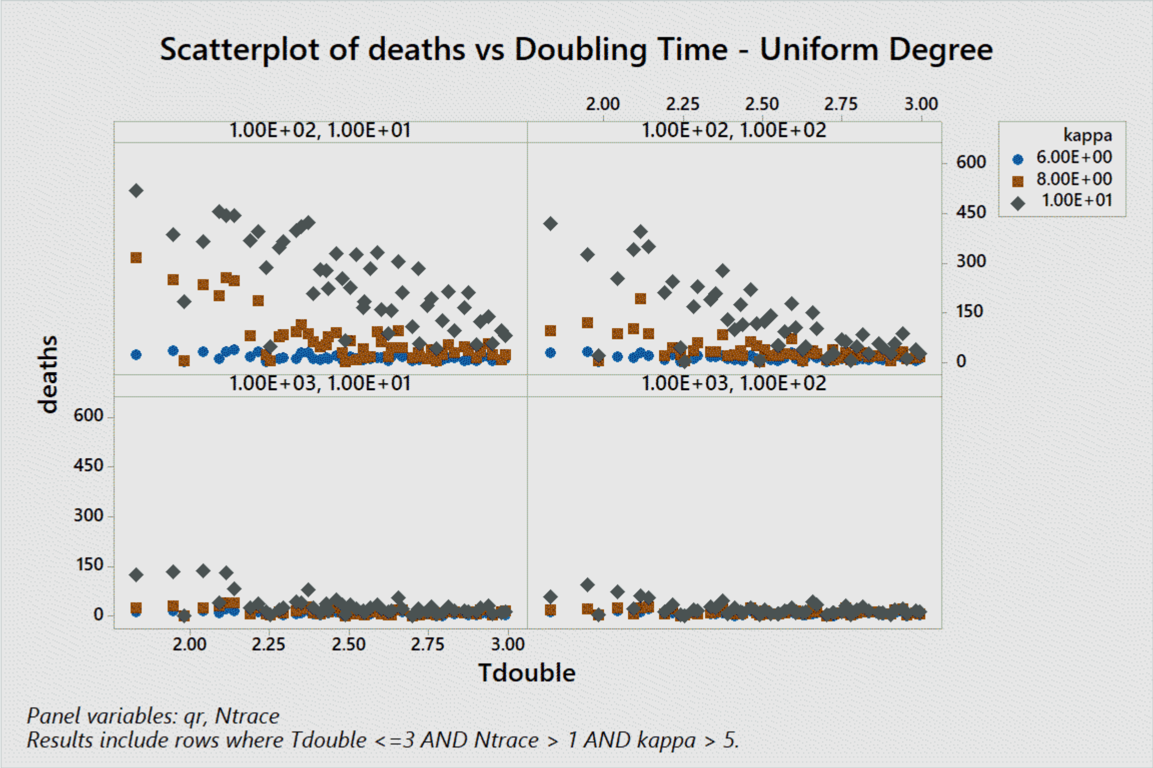

Figure 5-4. Stochastic Network Model-derived deaths using uniform degree distributions.

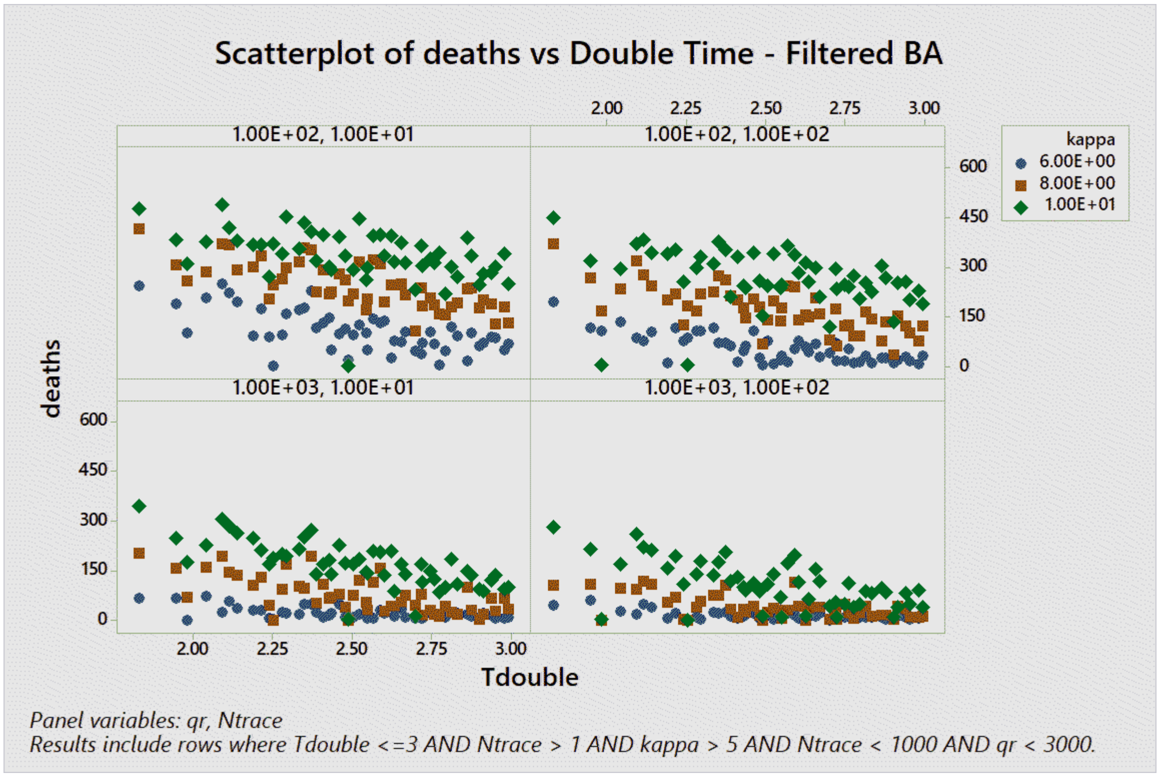

Figure 5-5. Stochastic Network Model-derived deaths using filtered BA networks.

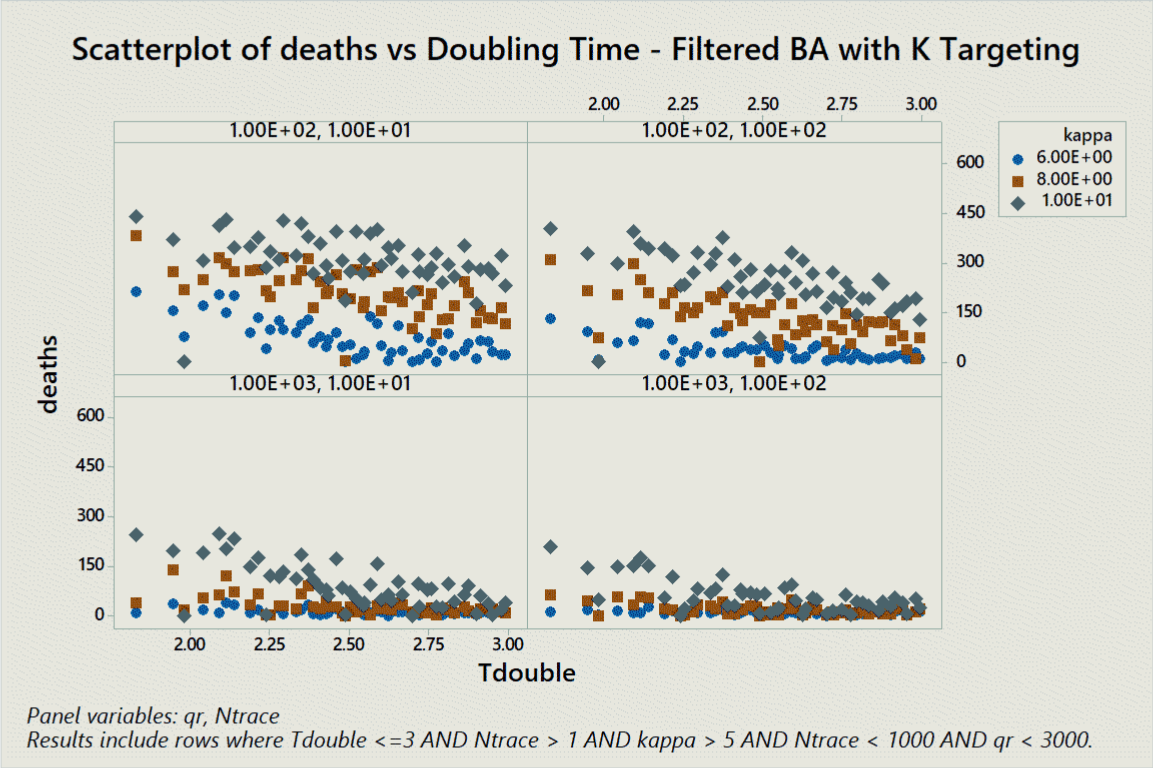

Figure 5-6. Stochastic Network Model-derived deaths using filtered BA networks with κ targeting.

6. CONTACT NETWORKS USING FOOT TRAFFIC DATA

As social distancing policy and recommendations went into effect across the nation, people made rapid changes to the places they visit and the way they interact. These changes are clearly seen in foot traffic data, which tracks mobility using location trackers in cell phones. Numerous news articles have documented trends in mobility as social distancing was put in place and then relaxed across different states (e.g. [12]). As part of this project, we acquired access to foot traffic data published by SafeGraph [16] which includes data on activity at over 5 million places across the US based on cell phone records.

In this analysis, SafeGraph data was analyzed to identify trends in behavior and to construct metrics designed to identify locations that may have an especially strong potential to foster disease transmission, either because of activity level or because visitors tend to come from many different locations. In addition, data on arrival rates, times, and durations have been used to construct contact networks for the Stochastic Network Model. This section provides a brief summary of the SafeGraph data and its analysis. Klise et al. [7] provides detailed methods and outcomes.

SafeGraph provides weekly data updates of the data in aggregate mobility patterns. SafeGraph anonymizes the data by applying noise, omitting data associated with a single mobile device, and grouping traffic according to the home census block group (CBG) of the mobile devices. Using this data, we build contact networks which store the interaction strength and transmission paths that can be used to study contact tracing and testing as well as disease transmission. This information is then used to study the impact that targeted business closures, restrictions, and use of PPE can have on person-to-person transmission.

A combination of SafeGraph weekly aggregate patterns data and open census data was used to create contact networks. This includes information on the number of devices that enter the POI on an hourly basis, a distribution of dwell times, and the device's home CBG. While the full dataset includes CBGs and POIs across the US, in practice, a region of interest or specific NAICS codes are used to downselect the data used in analysis. The current analysis focuses on individual counties. Bernalillo County in New Mexico is used to demonstrate the methods.

Mobility data can be analyzed to gain insights into many of the processes influencing disease spread. Geographic transmission can be fostered when the vistors to a location come from a wide range of home CBGs. The geographic diversity of a location's visitors can be measured using a bipartite graph, as described below. While the SafeGraph data is discretized using CBGs and POIs, these methods can be used at other scales. For example, if finer resolution data is available, the contact network could define nodes at the household level.

Contact networks store edge weights related to the interaction strength between individuals. The interaction strength is a function of concurrent visits to the same place and time spent at home. To use the Safegraph data, device counts are first scaled to number of people using the number of devices in the dataset and census information at the CBG scale. Sampling methods were also developed to extract representative patterns in this data. This ensures that the contact network remains a reasonable size while preserving the foot traffic patterns in the region of interest. Arrival time and dwell times were then sampled

from distributions established for each POI and visitors were assigned to their home CBG. This analysis results in a contact network where the edge weight is the interaction strength between nodes. This network that can be used in the Stochastic Network Model to determine the probability of local transmission between neighboring nodes and to model contact tracing. In this application, a node represents a typical individual from the CBG associated with the node.

The contact networks are analyzed using structural metrics such as the weighted node degree, shortest path length, and clustering. The weighted node degree, shown in Figure 6-1, results in an interaction strength for each node, or κ. The average weighted node degree, κ_*, is the average value across the network. The network metrics indicate that interactions clearly changed starting the week of March 9th. The decrease in weighted node degree indicates that people are in contact with less people as social distancing went into effect. An example network is shown in Figure 6-2. The node attribute is the weighted node degree and the link attribute is interaction strength. The small example illustrates strong links between a small number of nodes, and more frequent weaker links. More information is included in [7].

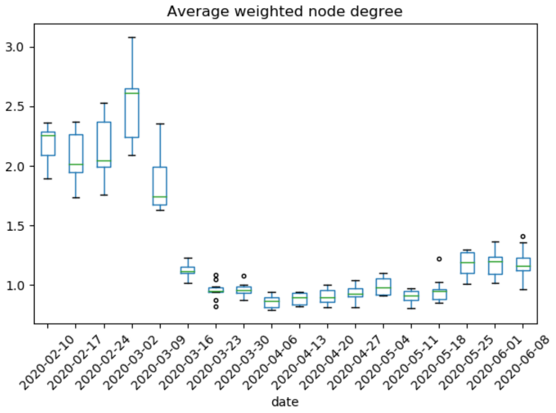

Figure 6-1. Weighted node degree between February and June for Bernalillo County.

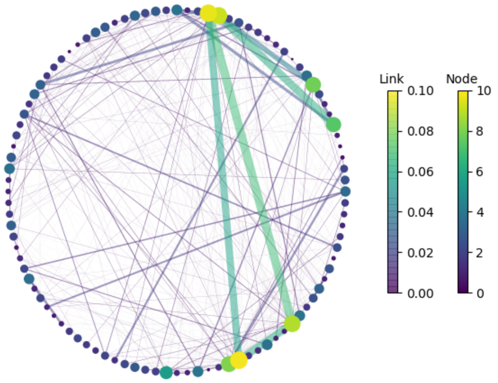

Figure 6-2. Subset of a contact network generated using mobility data for Bernalillo County showing interaction strength between nodes (link attribute) and weighted node degree (node attribute).

7. APPLICATIONS AND FUTURE DIRECTIONS

This project has developed a capability to help decision-makers navigate the trade-offs among available control strategies for COVID-19. The capability is designed to estimate the relative power of alternative controls to suppress outbreak. A summary and an example application is described below.

7.1. Overview and Approach

We use contact networks to study the effects of different levels of contact tracing, surveillance sampling, business closure (or modification to business practices sufficient to preclude transmission), and vaccine administration. These networks are designed to capture the role of specific kinds of locations as loci of disease spread, and to evaluate the power of control measures focused on location type. Simulations are not meant to track the specific history of the outbreak in each region because observed case counts depend on many contingent factors not included in this model and less relevant for designing disease suppression regimes. The example application described in this section focuses on Bernalillo County, New Mexico, however the capability has been applied to several other regions of the US.

We use SafeGraph mobility data from specific locations to learn how people gather and interact in different kinds of places. This data reflects local preferences and customs, along with general characteristics that might be similar in small towns and large cities. We apply this data to understand the effects of individuals' local travel, leisure activities, and interactions with business to quantify potential opportunities for disease spread that occur during individuals' daily interactions. Our analysis of this data enables us to anticipate possible transmissions at particular kinds of places, and thus derive risk metrics for various classes of locations and/or for particular identified locations of concern. From this understanding of individuals' patterns of movement we further investigate how targeted policies to change contact patterns could limit opportunities for transmission This perspective would eventually enable us to define optimal control strategies and determine if these strategies vary from region to region, or are the same everywhere.

The purpose of the epidemiological simulations is not to attempt to account for past case histories, nor to attempt specific predictions about the course of the outbreak in a region based on mobility data. Actual transmission patterns are subject to too many contingencies and influenced by too many uncertainties to permit credible forecasting. The goal is rather to use interaction patterns consistent with the mobility data to test control strategies, and to identify strategies that appear to perform well despite the contingencies that characterize the real system and our uncertainties about its properties.

A control strategy's success entails changing transmission networks in a way that dampens spread with high probability. Testing whether a particular strategy is useful goes beyond doing a single model run and seeing if a simulated outbreak is contained. Instead, many repeated runs looking at different initiating events and contingencies must be explored to see whether we get suppression with high probability across a wide range of potential scenarios. A single model run that exhibits outbreak suppression provides no support for the potential reliability of the control strategy it represents, even if it matches every historical data point

exactly. Instead, only large scale testing of a candidate control strategy across a wide range of possible contingencies can inform planners and decision makers of the potential reliability of that strategy.

In this study, we test for outbreak control potential by assuming the network and contact tracing and testing system is static and in place for a year. We seed the network with infected cases and measure key performance metrics (e.g. deaths from disease, peak hospitalization) as the disease spread and control dynamics interact. These metrics are the basis for discriminating successful from unsuccessful designs. Though replication of historical conditions to date may seem appealing to discriminate potential performance of policies, exercising the candidate policy across all relevant exigencies enables us to confidently judge the utility of model outputs as a basis for recommending future actions. Historical outbreak examples are important for calibrating the model and for defining the analytical strategy necessary to adequately explore the multidimensional space of possible inputs. Historical case count data enable us to understand and bound the number of active cases anticipated for a given population, which has strong implications for the performance of control systems (as discussed above in Section 4). This influence can be captured by examining a range of initial conditions for the simulation.

Quantitative analysis outputs pertain to performance targets for control systems (e.g. limiting the maximum number of people in any location to 10; reaching 80% of listed contacts within 24 hours and securing the cooperation of half with quarantine protocols). Outputs will show tradeoffs among control measures of this kind so that decision-makers can select appropriate combinations. The recommendations will come from finding systems that have a high probability of suppressing the disease.

We illustrate the capability using an analysis of data from Bernalillo County, New Mexico.

7.2. Process Steps

The process for assessing control system performance in a particular region begins with constructing contact networks for that region based on analysis of SafeGraph data. A sequence of weekly visit records, beginning with weeks prior to adoption of movement control policies and extending through subsequent weeks, generally including weeks of shut-down, were used to construct a corresponding series of contact networks. Each network comprised 10,000 individuals whose interconnections were determined by simulating arrival and departure events at specific locations based on the registered mobility data (see [7] for details). Network connections were constructed to be representative of those in a subset of the region's population, which is generally much larger than the modeled population.

For data collected over a given week, several alternative contact networks were constructed in order to capture uncertainties in the interpretation of the data in the context of the model. First, the full set of locations measured for the region of interest were sampled and scaled in order to produce a subset appropriate for the modeled population. This process was repeated to explore sample variability. Second, the differential propensity for contacts at different kinds of locations to transmit disease is not well known. Three alternatives having different degrees of variability among locations (denoted "constant", "linear", and "quadratic" below) were considered to assess the role of this uncertainty on policy performance.

The contact networks derived from weeks prior to mobility controls were then used to estimate the value of a single transmission probability parameter β, based on the assumption that COVID-19 would have an initial doubling time of T_d days under those conditions. The population average contact density in a pre-lockdown network (κ^*) is assumed to be associated with an unmitigated doubling time of T_d. The beta

value corresponding to this doubling time is then:

$$\beta^* = \frac{ln(2)}{T_d \kappa^*} \tag{7.1a}$$

This single calibration parameter enables disease spread to be simulated using the contact networks derived from each analyzed week. Each network might be used in turn to simulate disease spread for one week, with the aim of simulating a possible history for the real outbreak. This might be a useful verification exercise. In this kind of simulation, the effect of contact patterns in a given week would be integrated with the effects of prior weeks via the spread of cases passed on to the following week's network. The specific effect of each weeks' contact structure on disease spread is therefore impossible to discern. To measure the propensity for each week's pattern to amplify or suppress disease spread, we instead used each pattern separately as the basis for a one-year simulation in which the contact pattern persisted unchanged. This analysis lets us understand whether the patterns observed in each week would tend to contain the outbreak, or instead permit it to grow, if they were followed persistently.

This kind of analysis does not try to match a historical trajectory, but it can be tested against observations. Networks derived from weeks subject to mobility controls, during which a region's case counts were observed to decline, should show a small probability of outbreak spread. Figure 7-1 for example shows results derived for Bernalillo County. A stay-at-home order was issued for New Mexico on March 11. Visit data from the weeks of February 10 through March 9 were used to estimate κ^* for each of three assumptions about contact intensity variation. The corresponding β^*s were then used to simulate disease spread in each of those weeks as well as the following 13 weeks to June 8.

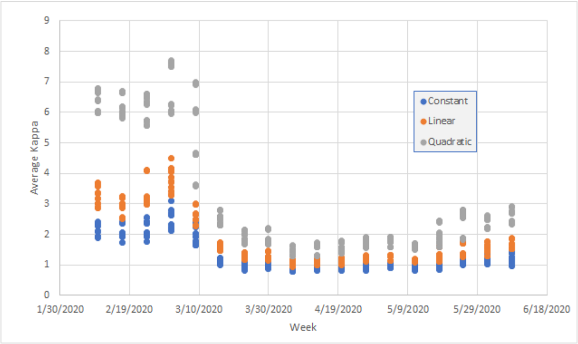

Figure 7-1. Average weighted contact-hours per hour based on location data in Bernalillo County, NM between February 24 and June 8, 2020, including three assumptions about variability in contact intensity over location types, and random samples.

Figure 7-2 summarizes the results from one-year simulations based on the contact networks derived from activity levels in different weeks. The Y axis shows total disease-induced fatalities at the end of the simulated year. Over this time the disease will generally either spread until a substantial fraction of the population has been infected, resulting in a total mortality of approximately 5% of the population, or will be confined to a subset of the population due to contact reduction. Total fatalities are therefore a good indicator of the tendency for the observed activity patterns, if they were followed habitually, to either foster or suppress disease spread. As discussed above, there are multiple simulations for each week corresponding to different random samples and to alternative assumptions about the kinds of contacts occurring at different types of location. The results are consistent with experience: the social distancing measures adopted in New Mexico from mid-March forward were generally effective in suppressing growth of the outbreak.

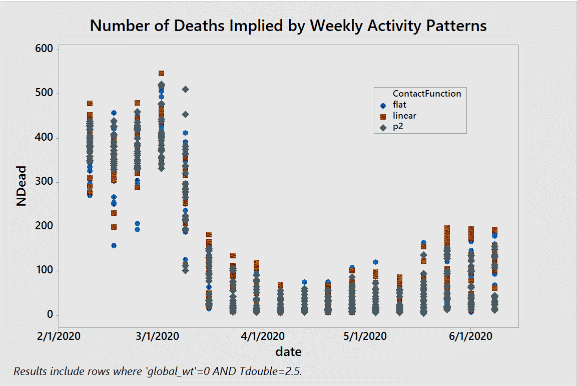

Figure 7-2. Number of disease-induced fatalities assuming the contact patterns derived for each week persisted until herd immunity or disease extinction.

Figure 7-2 does *not* show estimated weekly fatalities over the historical period: it shows whether the behavior observed in each week leads to contact networks that support outbreaks - and therefore high numbers of deaths - or containment. In general, networks conducive to disease spread and suppression should correspond to historical periods of increasing and decreasing case counts respectively. When they do, that confirms our assumption that the contact networks derived from activity data capture features of the real system that govern disease spread. This confirmation supports the use of such contact networks as a test-bed for other control measures.

7.3. Evaluation of Controls

Some control actions (such as frequent surveillance sampling and encouragement to wear masks) can be modeled as changes to epidemiological parameters that leave the pattern of contacts unchanged. Others (such as complete or partial contact reduction at specific kinds of establishments) lead to changes in the network structure. Parameter changes and structure changes are treated differently in the simulation workflow.

To understand the potential value of reducing contacts at specific kinds of locations, either by closing them or altering operational parameters that effectively eliminate the prospect of transmission, we defined three contact reduction scenarios based on the NAICS ids of affected facilities:

1 No closure or control representing the pre-pandemic status quo

2 Closing businesses in 20 of the 1058 digit NAICs classifications that tend to be associated with high contact density based on analysis of mobility data.

3 Closing a more limited subset of eight business types having the highest contact densities.

Our process for comparing outcomes from these three example policy alternatives recognizes that epidemiological effects of taking a particular control action are affected by many uncertainties, such as model parameters describing disease transmission processes, and compliance with imposed controls (e.g. willingness to wear masks as mandated). To rigorously assess potential control effectiveness we represent these uncertainties in terms of a range of action consequences.

We illustrate this process carrying forward the analysis of data from Bernalillo County. Key parameters describing the disease and control processes are summarized in Table 7-1.

Epidemiological/Uncontrollable Parameters

Parameter	Description	Dimensions	Value Range	Explanation/Sources
Contact Matrix	Matrix describing fraction each individual spends in a (standardized) contact situation with another	Contact time per unit time		Analysis of SafeGraph visit data
β	Probability of infection per unit time of contact	1/time	0.03 - 0.14	Inferred from analysis of contact network and assumption of rate of unmitigated spread (see text)
ε_p	Effectiveness of general PPE use in blocking transmission	1	0.2 - 0.8	UF study adopts 0.5, cites 20% - 80% for cloth masks
η_{ia}	Relative infectivity of contacts with asymptomatic infectious	1	0.25 - 0.75	Assumption

Policy/Control Parameters

Parameter	Description	Dimensions	Value Range	Explanation/Sources
Contact Matrix	Matrix describing fraction each individual spends in a (standardized) contact situation with another	Contact time per unit time		Analysis of SafeGraph visit data with location-dependent elimination of contacts for the contact reduction scenarios
q_r	Rate of random surveillance sampling	1/time (samples/day)	1,10,100,1000	Design parameter
N_{trace}	Number of contact tracers available	1	1,10,100	Design parameter
f_{vac}	Fraction of the population vaccinated in the first week	1	0, 0.007	Design parameter
r_{vac}	Rate of increase in vaccine availability	fraction/week	0,0.481	Design parameter
v_{eff}	Vaccine effectiveness in conferring immunity	fraction	0.5	Design parameter
v_{target}	Vaccine prioritization option - vaccination probability is proportional to connection strength	boolean	no, yes	Design parameter
x_{mask}	Mask wearing option - mask use us advocated in all public interactions	boolean	no, yes	Design parameter

Table 7-1. Epidemiological Parameter Variance for Sensitivity Study

Hundreds of thousands of combinations of parameter values were simulated, using contact networks derived from February 10 visit data as a baseline for unmitigated activity, with contracts at some locations suppressed for the contact reduction scenarios. The effects of modeled controls and their interactions can be understood by looking at the distribution of consequence measures for different settings of the relevant parameters. Figure 7-3 shows the distribution of the calculated number of fatalities for different values of the parameters governing surveillance sampling (q_r), contact tracing (N_{trace}), and mask-wearing (r_{mask}) for the network with no contact reduction. Ineffective control results in infection of a substantial fraction of the population with an attendant mortality of approximately 500. A successful strategy can reduce this significantly and with high probability. To achieve this outcome with no contact reduction, some combination of contact tracing (large N_{trace}), surveillance sampling (large q_r) and mask-wearing is evidently required. This conclusion holds for each of the assumptions regarding variation in contact density with location (columns on Figure 7-3).

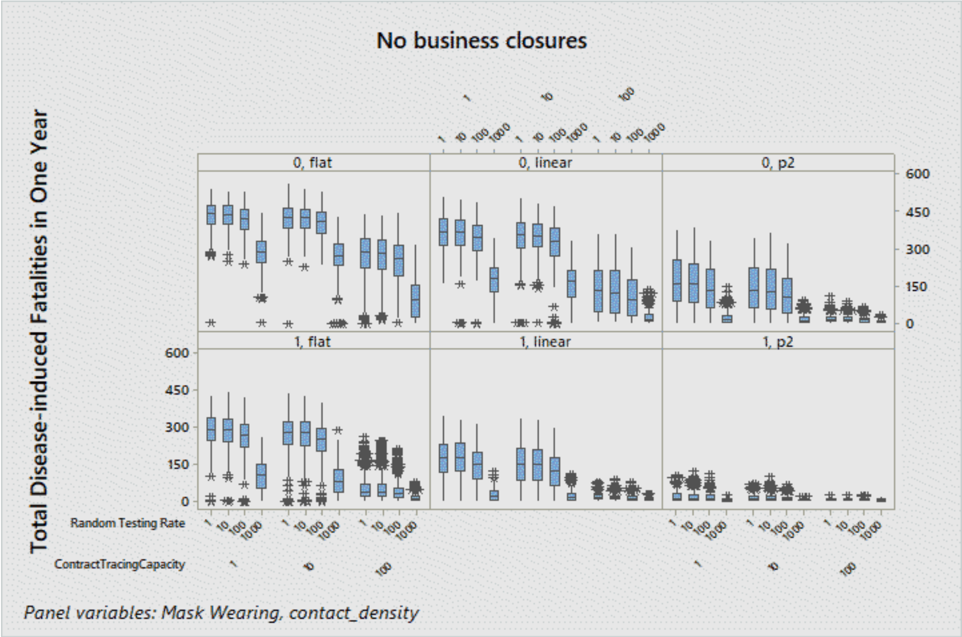

Figure 7-3. Distributions of modeled fatalities after one year for various combinations of control parameter values: No business closures

Figure 7-4 shows distributions of fatalities under the low contact reduction scenario, involving controls on 8 high-contact location types. This scenario suggests that consistently low fatalities might be reliably achieved with intensive contact tracing and surveillance sampling without general mask use, or alternatively that general use of masks would allow less intensive use of contact tracing and surveillance.

Imposing additional closures further relaxes requirements on the remaining control measures as shown in 7-5. In the absence of general mask-wearing (upper row) a high rate of surveillance sampling remains important for insuring containment.

These results illustrate the way in which newly-available information on mobility can inform designs for strategies to contain COVID-19 outbreaks with high reliability. Analysis of the model can provide insights into the parameters (such as disease prevalence in the population) and features (such as clustering in the contact network) that can have a strong influence on the performance of containment strategies.

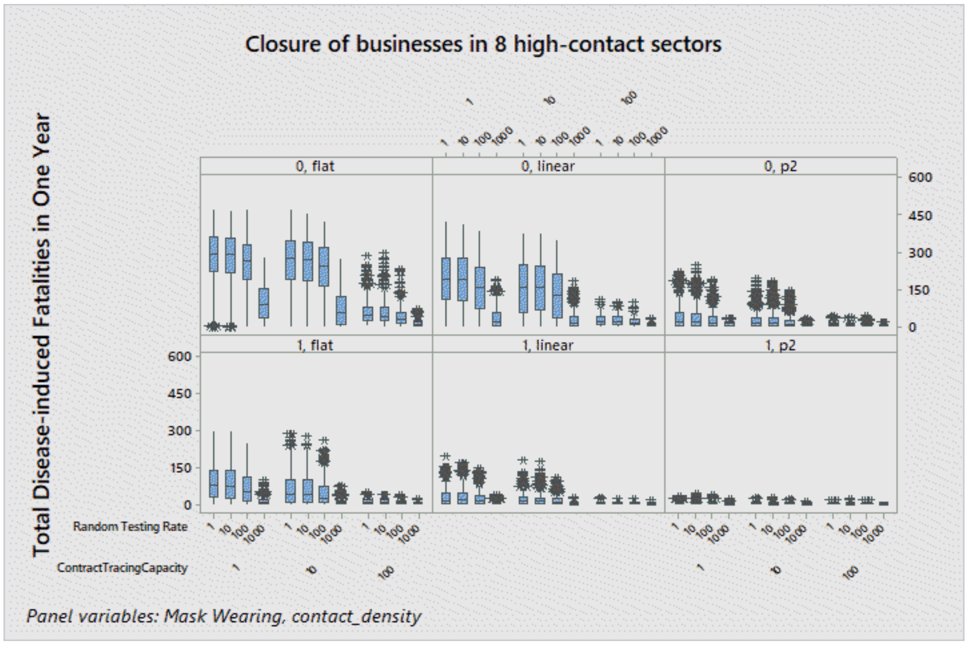

Figure 7-4. Distributions of modeled fatalities after one year for various combinations of control parameter values: Closure of businesses in 8 high-contact sectors

7.4. Summary and Future Directions

This work has created an analytical resource for informing urgent decisions regarding the design of NMI systems which are tailored to the specific conditions and constraints confronting public health officials. The integrated analysis tool enables key questions to be answered in a way that reflects current uncertainties, and that can be quickly updated as our understanding of the disease and public responses improves. Questions that can currently be addressed because of the work described in this report include:

- What factors determine whether outbreaks can be controlled via contact tracing and surveillance sampling vs. contact reduction?
- What resources, in terms of numbers of surveillance samples and rate of contact tracing, are needed to reduce outbreak risk to some acceptable level?
- Can targeting surveillance, PPE policies, social distance guidelines, or other NMI based on location, type of commercial activity, or other factors significantly improve containment?
- Which locations or kinds of locations are the biggest contributors to contact formation?
- What kind of information (e.g. regarding behavioral responses, PPE effectiveness, disease characteristics) would be most useful for designing more effective policies?

This work has led to a process for bringing information on disease characteristics and population behavior to bear on urgent outbreak management decisions while conveying outstanding uncertainties. Reducing those uncertainties through additional research can lead to better decisions. The analyses produced through this process can help identify where better information about system processes would be most valuable in

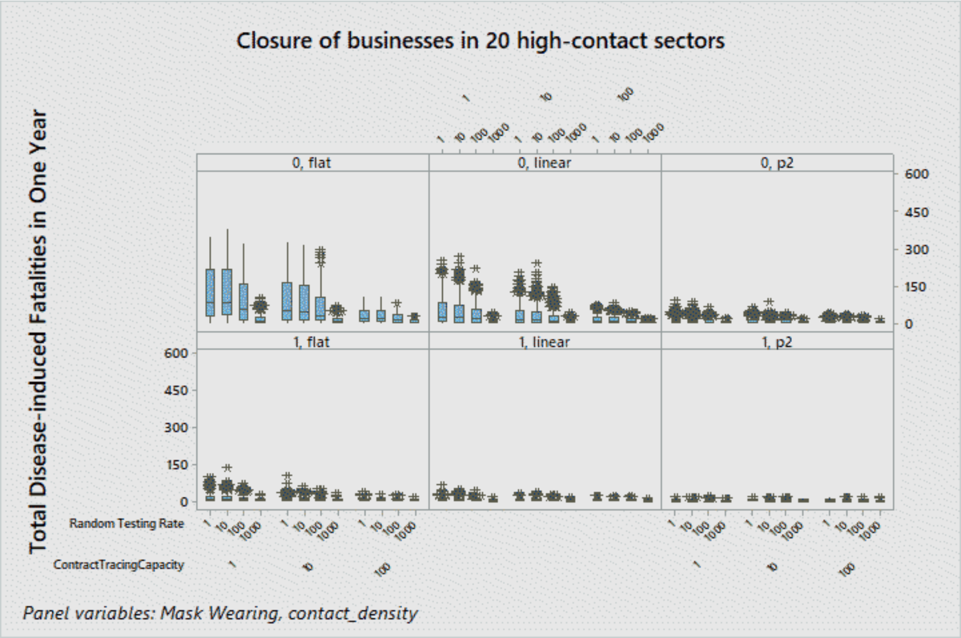

Figure 7-5. Distributions of modeled fatalities after one year for various combinations of control parameter values: Closure of businesses in 20 high-contact sectors

clarifying decisions. However, the process itself can benefit from improvements in methodology and data. Specific areas that might be pursued include:

- Analyses to-date have focused on a small number of study areas. Application to other regions and at different scales is straightforward given the national scope of the mobility data.
- Models for the effect of vaccines have been implemented but not analyzed in detail. Evaluation of the interaction of vaccine distribution with other mechanisms can help insure a low-risk relaxation of NMI measures as the proportion of immunized increases.
- Vaccination state model may need to be elaborated to represent multi-stage protocols or multiple vaccine types.
- Additionally, future directions for this model should include close collaboration with individuals and organizations actively engaged in disease control and mitigation. This close collaboration going forward will enable model developers and analysts to focus attention on the time critical questions most relevant during for ongoing disease control operations for which this model is uniquely poised to answer.

Additional research could provide information that would improve the utility and performance of this model:

- Relationship of contact frequency and duration on COVID-19 transmission is not well known in business and congregant settings. Better definition for the variability of contact intensity among location types would help improve accuracy.
- Behavioral changes are not represented. Closure of some businesses may cause increased traffic at

locations providing similar goods or services. A data-based model might be developed from responses seen in mobility data.

8. CONCLUSIONS

This report documents a compelling new approach to designing disease control policies that allocate scarce testing, contact tracing, and vaccination resources to better control community transmission of COVID19 or similar diseases. The Adaptive Recovery Model (ARM) combines a deterministic compartmental disease model with a stochastic network disease propagation model to enable us to simulate COVID-19 community spread through the lens of two complementary modeling motifs. ARM generates synthetic contact networks, leveraging cell-phone location data to identify locations within communities where the frequency and duration of interpersonal contacts create opportunities for rapid disease spread. ARM applies this model- and data-derived abstractions of community transmission to model the effectiveness of disease control measures including targeted social distancing, contact tracing, testing and vaccination. The architecture of ARM provides a unique capacity to support decision makers in understanding how best to deploy scarce testing, tracing and vaccination resources to minimize disease-spread potential in a community.

ARM was designed and developed over a very short time during the COVID-19 Pandemic. Fielding the model during the pandemic required that Sandia mathematicians and scientists design and implement major extensions to the state of the art for compartmental and network disease models, and create other capabilities such as data-driven contact network generation from scratch. The product provides a unique set of capabilities that address the needs of local, state/tribal, and national public health agencies to maximize the public health benefit achievable with limited availability of testing, tracing, and vaccination. This document details the novel mathematical formulations underlying the unique capabilities of ARM, dynamical stability analysis of the deterministic model components, sensitivity analysis of derived contact networks, and detailed derivation of contact networks from cell-phone location data. In addition, this report documents extensive High Performance Computing (HPC) based parameter studies that were run on the ARM model to analyze model and parameter uncertainty and develop defensible confidence intervals for model performance.

While the design and implementation of ARM is unique, its true value is in providing rigorous comparative analyses of disease control policies that maximize public health benefit from limited resources. This document also steps through applying ARM to evaluate three targeted social distancing policies using Bernalillo County, New Mexico as an exemplar test locale. This step-by-step analysis demonstrates the experimental design and analytical processes to be followed to confirm the relative performance of competing public health policies. Lastly this document explores a range of follow on activities to further exploit the power of ARM, along with additional research and development activities which could further expand capabilities and/or improve the generality of analytical results.

The documented design considerations, implementation details, and performance characteristics of the ARM model clearly demonstrate the potential utility of disease control models that provide decision makers with insights on effective allocation of chronically limited resources during an outbreak. Initial scenario tests of ARM shows that ARM's design focus on resource utilization rather than simple incidence prediction can provide decision makers with additional quantitative guidance for planning for and managing ongoing public health emergencies

REFERENCES

[1] Alberto Aleta, David Martin-Corral, Ana Pastore y Piontti, Marco Ajelli, Maria Litvinova, Matteo Chinazzi, Natalie E Dean, M Elizabeth Halloran, Ira M Longini, Stefano Merler, et al. Modeling the impact of social distancing, testing, contact tracing and household quarantine on second-wave scenarios of the covid-19 epidemic. *medRxiv*, 2020.

[2] Josh A Firth, Joel Hellewell, Petra Klepac, Stephen M Kissler, Adam J Kucharski, Lewis G Spurgin, CMMID COVID-19 working group, et al. Combining fine-scale social contact data with epidemic modelling reveals interactions between contact tracing, quarantine, testing and physical distancing for controlling covid-19. *medRxiv*, 2020.

[3] Robert J Glass, Laura M Glass, Walter E Beyeler, and H Jason Min. Targeted social distancing designs for pandemic influenza. *Emerging infectious diseases*, 12(11):1671, 2006.

[4] Scott Gottlieb, C Rivers, M McClellan, L Silvis, and C Watson. National coronavirus response: a road map to reopening. *American Enterprise Institute*, 29, 2020.

[5] Joel Hellewell, Sam Abbott, Amy Gimma, Nikos I Bosse, Christopher I Jarvis, Timothy W Russell, James D Munday, Adam J Kucharski, W John Edmunds, Fiona Sun, et al. Feasibility of controlling covid-19 outbreaks by isolation of cases and contacts. *The Lancet Global Health*, 2020.

[6] Herbert W Hethcote. The mathematics of infectious diseases. *SIAM review*, 42(4):599–653, 2000.

[7] Katherine Klise, Walt Beyeler, Patrick Finley, and Monear Makvandi. Analysis of mobility data to build contact networks for COVID-19. *in preparation*, 0:0, Sep 2020.

[8] Kin On Kwok, Arthur Tang, Vivian WI Wei, Woo Hyun Park, Eng Kiong Yeoh, and Steven Riley. Epidemic models of contact tracing: Systematic review of transmission studies of severe acute respiratory syndrome and middle east respiratory syndrome. *Computational and structural biotechnology journal*, 2019.

[9] Maia Martcheva. *An introduction to mathematical epidemiology*, volume 61. Springer, 2015.

[10] Robert M. May and Alun L. Lloyd. Infection dynamics on scale-free networks. *Phys. Rev. E*, 64:066112, Nov 2001.

[11] McGee, R. S. Models of seirs epidemic dynamics with extensions, including network-structured populations, testing, contact tracing, and social distancing. https://github.com/ryansmcgee/seirsplus. Accessed: April 22, 2020.

[12] McMinn, Sean and Talbot, Ruth. Mobile phone data show more americans are leaving their homes, despite orders. https://www.npr.org/2020/05/01/849161820/mobile-phone-data-show-more-americans-are-leaving-their-homes-despite-orders. Accessed: June 4,

2020.

[13] Kamalich Muniz-Rodriguez, Gerardo Chowell, Chi-Hin Cheung, Dongyu Jia, Po-Ying Lai, Yiseul Lee, Manyun Liu, Sylvia K Ofori, Kimberlyn M Roosa, Lone Simonsen, et al. Early release-doubling time of the covid-19 epidemic by province, china. 2020.

[14] Calistus N Ngonghala, Enahoro Iboi, Steffen Eikenberry, Matthew Scotch, Chandini Raina MacIntyre, Matthew H Bonds, and Abba B Gumel. Mathematical assessment of the impact of non-pharmaceutical interventions on curtailing the 2019 novel coronavirus. *Mathematical Biosciences*, page 108364, 2020.

[15] Corey M Peak, Lauren M Childs, Yonatan H Grad, and Caroline O Buckee. Comparing nonpharmaceutical interventions for containing emerging epidemics. *Proceedings of the National Academy of Sciences*, 114(15):4023–4028, 2017.

[16] SafeGraph. Safegraph covid-19 data consortium. https://www.safegraph.com/covid-19-data-consortium. Accessed: June 4, 2020.

[17] Steven Sanche, Yen Ting Lin, Chonggang Xu, Ethan Romero-Severson, Nick Hengartner, and Ruian Ke. Early release-high contagiousness and rapid spread of severe acute respiratory syndrome coronavirus 2. 2020.

[18] D Siddarth and G Weyl. Why we must test millions a day. *COVID-19 Rapid Response Impact Initiative| White Paper*, 6, 2020.

DISTRIBUTION

Email—Internal (encrypt for OUO)

Name	Org.	Sandia Email Address
Justin Garretson	6617	jrgarr@sandia.gov
Sean DeRosa	8721	sderosa@sandia.gov
Courtney S. Dornburg	5954	ccdornb@sandia.gov
Stephanie P. Kuzio	8825	spkuzio@sandia.gov
Patrick Finley	8721	pdfinle@sandia.gov
Technical Library	01177	libref@sandia.gov

Sandia National Laboratories

Sandia National Laboratories is a multimission laboratory managed and operated by National Technology & Engineering Solutions of Sandia LLC, a wholly owned subsidiary of Honeywell International Inc., for the U.S. Department of Energy's National Nuclear Security Administration under contract DE-NA0003525.
